# Phenotypic spectrum of the first Belgian *MYBPC3* founder: a large multi-exon deletion with a varying phenotype

**DOI:** 10.3389/fgene.2024.1392527

**Published:** 2024-05-21

**Authors:** Hanne M. Boen, Maaike Alaerts, Lut Van Laer, Johan B. Saenen, Inge Goovaerts, Jarl Bastianen, Pieter Koopman, Philippe Vanduynhoven, Elke De Vuyst, Michael Rosseel, Hein Heidbuchel, Emeline M. Van Craenenbroeck, Bart Loeys

**Affiliations:** ^1^ Research Group Cardiovascular Diseases, Genetics, Pharmacology and Physiopathology of Heart, Blood Vessels and Skeleton (GENCOR) Department, University of Antwerp, Antwerp, Belgium; ^2^ Department of Cardiology, Antwerp University Hospital, Antwerp, Belgium; ^3^ Center of Medical Genetics, Cardiogenomics, Genetics, Pharmacology and Physiopathology of Heart, Blood Vessels and Skeleton (GENCOR) Department, Antwerp University Hospital and University of Antwerp, Antwerp, Belgium; ^4^ Hartcentrum Hasselt, Jessa Hospital Hasselt, Hasselt, Belgium; ^5^ Department of Cardiology, ASZ Aalst, Aalst, Belgium

**Keywords:** *MYBPC3*, founder variant, cardiogenetic, phenotyping, hypertrophic cardiomyopathy

## Abstract

**Background:**

Variants in the *MYBPC3* gene are a frequent cause of hypertrophic cardiomyopathy (HCM) but display a large phenotypic heterogeneity. Founder mutations are often believed to be more benign as they prevailed despite potential negative selection pressure. We detected a pathogenic variant in *MYBPC3* (del exon 23-26) in several probands. We aimed to assess the presence of a common haplotype and to describe the cardiac characteristics, disease severity and long-term outcome of mutation carriers.

**Methods:**

Probands with HCM caused by a pathogenic deletion of exon 23-26 of *MYBPC3* were identified through genetic screening using a gene panel encompassing 59 genes associated with cardiomyopathies in a single genetic center in Belgium. Cascade screening of first-degree relatives was performed, and genotype positive relatives were further phenotyped. Clinical characteristics were collected from probands and relatives. Cardiac outcomes included death, heart transplantation, life-threatening arrhythmia, heart failure hospitalization or septal reduction therapy. Haplotype analysis, using microsatellite markers surrounding *MYBPC3*, was performed in all index patients to identify a common haplotype. The age of the founder variant was estimated based on the size of the shared haplotype using a linkage-disequilibrium based approach.

**Results:**

We identified 24 probands with HCM harbouring the *MYBPC3* exon 23-26 deletion. Probands were on average 51 ± 16 years old at time of clinical HCM diagnosis and 62 ± 10 years old at time of genetic diagnosis. A common haplotype of 1.19 Mb was identified in all 24 probands, with 19 of the probands sharing a 13.8 Mb haplotype. The founder event was estimated to have happened five generations, or 175–200 years ago, around the year 1830 in central Flanders. Through cascade screening, 59 first-degree relatives were genetically tested, of whom 37 (62.7%) were genotype positive (G+) and 22 (37.3%) genotype negative (G-). They were on average 38 ± 19 years old at time of genetic testing. Subsequent clinical assessment revealed a HCM phenotype in 19 (51.4%) G+ relatives. Probands were older (63 ± 10 vs. 42 ± 21 years; *p* < 0.001) and had more severe phenotypes than G+ family members, presenting with more symptoms (50% vs. 13.5%; *p* = 0.002), arrhythmia (41.7% vs. 12.9%, *p* = 0.014), more overt hypertrophy and left ventricular outflow tract obstruction (43.5% vs. 3.0%; *p* < 0.001). Male G+ relatives more often had a HCM phenotype (78.6% vs. 34.8%; *p* = 0.010) and were more severely affected than females. At the age of 50, a penetrance of 78.6% was observed, defined as the presence of HCM in 11 of 14 G+ relatives with age ≥50 years. Overall, 20.3% of all variant carriers developed one of the predefined cardiac outcomes after a median follow-up of 5.5 years with an average age of 50 (±21) years.

**Conclusion:**

A Belgian founder variant, an exon 23-26 deletion in *MYBPC3*, was identified in 24 probands and 37 family members. The variant is characterized by a high penetrance of 78.6% at the age of 50 years but has variable phenotypic expression. Adverse outcomes were observed in 20.3% of patients during follow-up.

## 1 Introduction

As the evidence of a genetic basis in hypertrophic cardiomyopathy (HCM) has grown in the last decades, genetic testing in all patients with HCM is now recommended by both the American Heart Association and the European Cardiac Society (Musunuru et al., 2020; Wilde et al., 2022). In about half of all patients a genetic diagnosis can be made, with a diagnostic yield of about 32%–40% in unselected patients and 70% in patients with a positive family history (Alfares et al., 2015; Wilcox and Hershberger, 2018). In an additional 9%–15% of patients a variant of uncertain significance (VUS) is identified (Alfares et al., 2015). Causal genetic variation in HCM patients is most often found in the sarcomeric genes (Van Driest et al., 2005; Wilcox and Hershberger, 2018), including *MYBPC3*, which is the most frequently affected gene, with up to 30%–57% of all identified variants (Richard et al., 2003; Alfares et al., 2015).

The myosin-binding protein C (MyBP-C) protein is part of the thick filament of the sarcomere (Winegrad, 1999). The specific cardiac isoform, cMyBP-C can be found in cardiomyocytes only and is encoded by the *MYBPC3* gene, located on chromosome 11 and consisting of 35 exons (Carrier et al., 1997; Winegrad, 1999). The cMyBP-C-protein binds to myosin with its C-terminal module as well as to titin, another important structural protein of the sarcomere (Winegrad, 1999). Overall, cMyBP-C plays a regulatory role in cardiac contractility by altering myofilament sliding velocity (Winegrad, 1999).

Both missense and loss-of-function variants in *MYBPC3* have been associated with HCM (Walsh et al., 2017; Ingles et al., 2019). Truncating variants in *MYBPC3* can be considered pathogenic since they associate strongly with HCM and haploinsufficiency is a well-established disease mechanism (Walsh et al., 2017). Copy Number Variants (CNV) involving *MYBPC3* are seen in 1.4% of HCM patients (Mates et al., 2020). Depending on the size and exact location, deletions can lead to a premature stop-codon, leading to truncation, or result in a shortened protein (Rottbauer et al., 1997; Andersen et al., 2004; Frank-Hansen et al., 2008).

In HCM, an extensive phenotypic variability in severity and outcomes is seen. The identification of a sarcomeric mutation seems to play a role in disease severity as genotype positive HCM patients have a two-fold higher risk for adverse outcomes (death, heart failure, malignant ventricular arrhythmias, and atrial fibrillation) than genotype negative HCM patients (Ho et al., 2018). Interestingly, also HCM patients with a VUS show an increased incidence of death and atrial fibrillation compared to completely genotype negative HCM patients (Ho et al., 2018). The specific affected gene also influences disease severity and outcome, with *MYH7* carriers being in general more severely affected compared to *MYBPC3* carriers (Ho et al., 2018).

In addition, genetic and environmental modifiers, such as arterial hypertension, likely play a role in this observed phenotypic variability (Daw et al., 2007; Claes et al., 2016; Walsh et al., 2017; Helms et al., 2020).

Within the group of *MYBPC3*-variant harbouring individuals a significant degree of phenotypic variability is observed, with variable penetrance and expressivity, depending on age and gender (Page et al., 2012; Helms et al., 2020). Founder variants, generally present in large cohorts, provide interesting opportunities to investigate genetic and non-genetic modifiers. This results in a better understanding of phenotype-genotype correlations which can aid in genetic counselling of patients and family members.

Here, we describe the phenotypic spectrum of an exon 23-26 deletion in *MYBPC3*, a Belgian founder variant.

## 2 Materials and methods

### 2.1 Proband selection and variant identification

In a total of 1,364 HCM probands who presented at the cardiogenetics clinic of the Antwerp University Hospital for genetic testing, from January 2015 until October 2023, a recurrent *MYBPC3* (ENST00000545968; NM_000256.3) variant, a large heterozygous deletion of exon 23 to 26 (del23-26), was identified in 24 distinct families (1.8% of probands). Probands were defined as patients with a new diagnosis of HCM, presenting for cardiogenetic diagnosis, without prior knowledge of a genetic variant within their family. For each proband a family tree was drawn up to four generations. Through comparison of family trees, possible closely related probands could be identified, This was the case for two separately presenting individuals of family three, which were subsequently merged in 1 single family with the first identified patient serving as proband.

This cardiogenetic screening was performed using a cardiomyopathy (CM) next-generation sequencing (NGS) gene panel encompassing 59 genes associated with CMs. In short, genomic DNA of HCM patients was extracted from EDTA blood using standard procedures (Chemagic DNA bloodkit special, Perkin Elmer, Waltham, MA, United States). Genetic analysis was performed using either custom HaloPlex target enrichment followed by NGS (Illumina) as previously described (Proost et al., 2015) (before June 2021) or whole exome sequencing after enrichment of the exome (Twist Human Core Exome kit, Twist Bioscience with additional custom probes) (starting from June 2021). The deletion was identified through CNV analysis using a read-depth strategy (SeqPilot, JSI) and confirmed with a multiplex amplicon quantification (MAQ) assay. When applying a read-depth strategy for CNV analysis, the raw number of reads for each region of interest (ROI) is normalized against the number of reads of selected control regions within the sample and compared between all samples within the same sequencing run. A deletion is characterized by approximately 50% of the reads found when compared to controls, while a duplication will show approximately 150% of the reads.

### 2.2 Cascade screening

Cascade-screening of first-degree relatives was performed. Family members were invited to participate by family letter and genetic counselling by a multidisciplinary team was offered before and after variant testing. After family members underwent genetic testing (using MAQ) for the *MYPBC3* del23-26 variant, genotype positive (G+) relatives were subsequently offered clinical evaluation. If the proband carried an additional variant, identified with the 59 CM-panel, this additional variant was sequenced in the family members as well, using sanger sequencing.

### 2.3 Clinical data

Demographic characteristics, cardiac assessment and outcome were collected from probands and relatives’ patient files. Composite cardiac outcome included death, heart transplantation (HTx), life-threatening arrhythmia, heart failure hospitalization or septal reduction therapy.

### 2.4 Identification of the deletion break point

To assess the exact localization of the breakpoints of the deletion a touchdown PCR was performed using a forward primer located at the end of exon 22 and a reverse primer at the beginning of exon 27 of the *MYBPC3* gene (95°C 5′, 20× (95°C 30″, 65 (−0.5×)°C 30″, 72°C 1′), 15× (95°C 30″, 55°C 30″, 72°C 1′), 72°C 10′, 15°C 10′).

*MYBPC3*-E22F-M13F: GTT​TTC​CCA​GTC​ACG​ACG​AAT​AAG​GCC​CCA​GCC​AGG​CCA

*MYBPC3*-E27R-M13R: CAG​GAA​ACA​GCT​ATG​ACA​TGT​GTG​CTC​TGT​CAG​CCC​CTG​CA

The presence of a PCR product within the expected range was confirmed on Qiaxcel^®^ capillary electrophoresis (Qiagen). For the non-affected allele, we did not expect a PCR product as this would be too big (>4,000 bp) for amplification. Sanger sequencing was used to sequence the resulting PCR product (3500 Dx Genetic Analyzer, Applied Biosystems) and sequence was compared to the reference sequence ENST00000545968.6 retrieved from Ensembl (Martin et al., 2022) using CLC DNA workbench software version 5.7.1 (CLC Bio, Aarhus, Denmark).

### 2.5 Haplotype analysis

**FIGURE 1:**
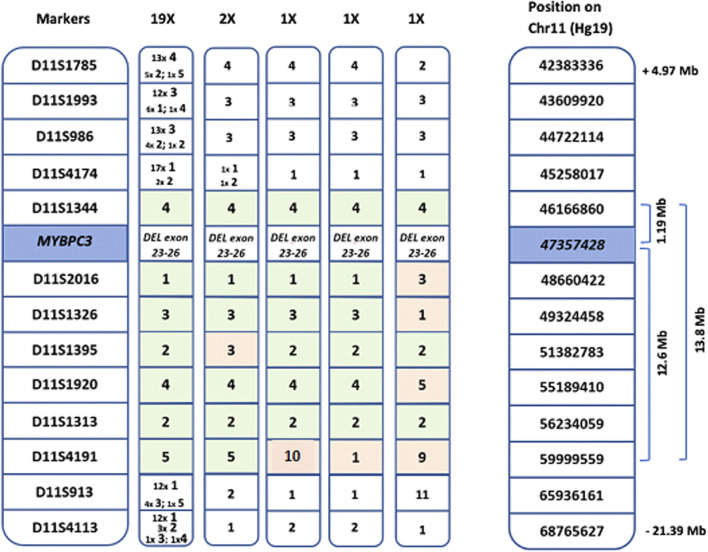
Length of the common haplotype, based on markers surrounding the *MYBPC3* gene. Shared haplotypes spanning seven markers derived from the 24 probands. From left to right: common haplotype found in 19 probands, 4 haplotypes of the 5 probands with recombination events and genomic location of the markers on chromosome 11 (Hg19). The common haplotype shared by all probands is 1.19 Mb, 19 of the 24 probands share a common haplotype of 13.8 Mb.

First, a shared genetic background was identified in all index patients, based on intragenic SNPs within the *MYBPC3* gene (Table 2). Next, haplotype analysis, using microsatellite markers surrounding *MYBPC3*, was performed to confirm the presence and assess the size of the common haplotype in all probands (Figure 1).

Thirteen microsatellite markers surrounding *MYBPC3* were analyzed in all selected probands as has been described before (Bergman et al., 2001) (D11S1785; D11S1993; D11S986; D11S4174; D11S1344; D11S2016; D11S1326; D11S1395; D11S1920; D11S1313; D11S4191; D11S913; D11S4113). Forward primers were labeled with 6-fluorescein amidite (FAM), and PCR products were analyzed in the presence of an internal sizing standard (ROX) using ABI3130XL (Applied Biosystems). The sizes of the amplicons were determined using ABI GeneMapper software v3.7 (Applied Biosystems).

After confirming a shared haplotype, the age of the variant was estimated based on the common microsatellite markers using a linkage-disequilibrium based approach (Bergman et al., 2001). The age of the variant (*t*, in number of generations) was estimated using the following formula: t=lnPd1−Pn21−Pn2ln1−r

Where Pd1 is the frequency of the founder marker allele in variant-carrying haplotypes and Pn2 is the frequency of the founder marker allele in non-variant carrying haplotypes. The founder marker allele was assumed to be the most frequent allele among the variant-carrying haplotypes. When the founder marker allele is present in all variant-carrying haplotypes (Pd1 = 1), but absent from all non-variant carrying haplotypes (Pn2 = 0), this formula can no longer be used as the estimated mutation age would equal 0. *r* is the recombination fraction between a marker and the variant and is calculated as: $r=\frac{1-e^{-2d}}{2}$ where d is the distance of the marker to the variant in cM/100. Distance of the shared haplotype in cM was based on the distance in Mb, assuming that 1 Mb ≈ 1 cM. Each generation was assumed to be 25 years when calculating the age of the variant. An estimation of mutation age for each tested marker was calculated, after which the average of estimations was used to derive the final estimation as has been reported before (Bergman et al., 2001).

### 2.6 Statistical analysis

Frequencies are expressed as numbers and percentages. Continuous variables are expressed as mean ± SD if normally or median ± interquartile range if non-normally distributed. Unpaired T-test or Mann-Whitney U test was used for comparisons of continuous variables. Chi-square test was used for comparisons of categorical variables. All analyses were performed using SPSS Statistics version 28 (IBM Corporation). A *p*-value of <0.05 was considered statistically significant.

## 3 Results

### 3.1 Identification of probands

From January 2015 till October 2023, we identified 24 probands with HCM carrying the *MYBPC3* exon 23-26 deletion (Table 1), with a mean age 51 ± 16 years at time of clinical HCM diagnosis and 62 ± 10 years at time of genetic diagnosis. A positive family history of HCM was present in ten (41.7%) probands, sudden cardiac death (SCD) had occurred in nine families (37.5%) of which in two (8.3%) families before the age of 35.

**TABLE 1:** Overview of patient characteristics and comparison between probands and G+ relatives.

	*MYBP3* del Exon 23-26			
	Overall	Proband	G+ relative	*p*-value
	*N* = 61	*N* = 24	*N* = 37	
Baseline characteristics				
Male gender (*n*, %)	27 (44.3)	13 (54.2)	14 (37.8)	0.210
Current age (y)	50 ± 21 [1–87]	63 ± 10 [50–87]	42 ± 21 [1–84]	**<0.001**
Age at clinical Dx (y)	50 ± 17 (*N* = 43)	51 ± 16	45 ± 21 (*N* = 19)	0.257
Age at genetic Dx (y)	49 ± 21	62 ± 10	40 ± 21	**<0.001**
Additional variant (*n*, %)^a^	22 (36.1)	11 (45.8)	11 (29.7)	0.201
Clinical characteristics				
HCM phenotype (*n*, %)	43 (70.5)	24 (100)	19 (51.4)	**<0.001**
Symptomatic at time of Dx (*n*, %)	17 (27.9)	12 (50)	5 (13.5)	**0.002**
Risk Factors	*N* = 58	*N* = 24	*N* = 34	
Arterial Hypertension (*n*, %)	26 (44.8)	12 (50)	14 (37.8)	0.506
Rhythm and Conduction disturbances	*N* = 55	*N* = 24	*N* = 31	
IV conduction disturbance (*n*, %)	14 (25.5)	7 (29.2)	7 (22.6)	0.578
Repolarization disturbances (*n*, %)	18 (50.9)	17 (70.8)	11 (35.5)	**0.009**
Atrial fibrillation (*n*, %)	14 (25.5)	8 (33.3)	6 (19.4)	0.238
nsVT (*n*, %)	14 (25.5)	10 (41.7)	4 (12.9)	**0.014**
Echocardiography	*N* = 56	*N* = 23	*N* = 33	
LVEF < 50% (*n*, %)	5 (8.9)	4 (17.4)	1 (3.0)	0.064
IVS (mm)	16.3 ± 5.5	20.6 ± 3.8	12.9 ± 4.1	**<0.001**
PWT (mm)	10.6 ± 2.6	12.4 ± 2.4	9.5 ± 2.1	**<0.001**
IVS/PWT (mm)	1.4 ± 0.4	1.1 ± 0.1	1.2 ± 0.2	**0.001**
LA dilatation (*n*, %)	25 (44.6)	16 (69.6)	9 (27.3)	**<0.001**
SAM (*n*, %)	9 (16.1)	7 (30.4)	2 (6.1)	**0.019**
LV Outflow tract obstruction provoked or at rest (*n*, %)	11 (19.6)	10 (43.5)	1 (3.0)	**<0.001**
Cardiac MRI	*N* = 20	*N* = 14	*N* = 6	
Cardiac mass (g)	153 ± 80	164 ± 66	103 ± 32	0.063
Indexed cardiac mass (g/m^2^)	85 ± 52	91 ± 36	56 ± 14	0.102
IVS (mm)	18.7 ± 5.1	19.5 ± 4.1	15.5 ± 7.4	0.163
Presence of LGE (*n*, %)	14 (70)	12 (85.7)	2 (33.3)	**0.019**
Pharmacological therapy	*N* = 57	*N* = 24	*N* = 33	
Beta blockade (*n*, %)	30 (52.6)	18 (75)	12 (36.4)	**0.004**
Calcium blocker (*n*, %)	11 (19.3)	5 (20.8)	6 (18.2)	0.802
RAAS blockers (*n*, %)	23 (40.4)	11 (45.8)	12 (36.4)	0.472
Diuretic therapy (*n*, %)	8 (14.0)	6 (25)	2 (6.1)	**0.042**
Outcomes	*N* = 59	*N* = 24	*N* = 35	
Duration of follow-up (y)	5.5 [1–22]	7.0 [1–22]	3.5 [1–20]	0.137
Cardiac death (*n*, %)	0	0	0	1
Heart transplant (*n*, %)	2 (3.4)	1 (4.2)	1 (2.9)	0.785
Life-threatening arrhythmia (*n*, %)	3 (5.2)	3 (12.5)	0 (0)	**0.034**
Heart failure hospitalization (*n*, %)	9 (15.5)	7 (29.2)	2 (5.9)	**0.016**
Septal reduction therapy (*n*, %)	4 (6.9)	3 (12.5)	1 (2.9)	0.143
Device (*n*, %)	7 (12.1)	6 (25.0)	1 (2.9)	**0.011**
Composite outcome (*n*, %)	12 (20.3)	8 (33.3)	4 (11.4)	**0.040**

^a^ G+ relatives were screened only for additional variants present in the proband and were not assessed for the full 59 genes CM panel. Del, deletion; Dx, diagnosis; G+ relative, genotype positive relative; HCM, hypertrophic cardiomyopathy; IV, intraventricular; IVS, intraventricular septum; LA, left atrium; LGE, late gadolinium enhancement; LV, left ventricle; LVEF, left ventricular ejection fraction, MRI, magnetic resonance imaging; nsVT, non-sustained ventricular tachycardia; PWT, posterior wall thickness; RAAS, renin-angiotensin-aldosterone system; SAM, systolic anterior motion of mitral valve; y, years. Bold values are significant differences.

### 3.2 Identification of the deletion breakpoint

Using a nested-PCR approach we delineated the exact breakpoints of the *MYBPC3* deletion of exon 23 to 26. The complete deletion spans 3546 base pairs from intron 22 to intron 26: Chr11:g.47360721 – 47357175del (GRCh37/hg19). Exon 23 and part of exon 24 encode the last part of the C5 immunoglobulin-I domain whereas the remainder of exon 24, exon 25 and exon 26 encode the C6 fibronectin-3 domain of the cMyBP-C protein (Carrier et al., 1997).

### 3.3 Variant classification

The variant was classified as pathogenic (class 5) according to ACMG guidelines (Richards et al., 2015; Riggs et al., 2020) with the following arguments: 1. Copy number loss content 1A (contains protein-coding functionally important element; 0p); 2. Overlap with established haplo-insufficient gene 2E [both breakpoints are within the same gene; classification according to sequence variant interpretation criteria: PVS1 (null variant in a gene where loss of function is a known mechanism), PS4/PM2 (increased prevalence in affected individuals compared to controls/absent from controls); PP1 (co-segregation with disease in multiple affected family members); 0.9 p]; and 3. Evaluation of number of genes in the CNV: 3A (0-24 genes; 0p); 4. Detailed evaluation of genomic content 4F and 5D (7 or more observed segregations; 0.45 p). 66 informative meioses were present over all families. Of a total of 20 relatives with HCM, 19 carried the *MYBPC3* variant. An overview of the pedigrees of all 24 families is available in the Supplementary Material.

### 3.4 Haplotyping

A common genetic background, defined by intragenic SNPs within the *MYBPC3* gene was identified in all 24 probands (Table 2).

**TABLE 2:** Overview of shared genetic background in probands. Based on the allelic frequency of the intragenic SNPs, within the *MYBPC3* gene. The chance of all 24 probands having the same haplotype by chance is calculated to be 2.07*10^25^, suggesting a founder event.

c. (ENST00000545968)	SNP I.D.	GRCh38 genomic position	Allelic frequency all (GnomAD v4)	Allelic frequency non-Finnish Europeans (GnomAD v4)
c.472G>A Present	rs3729986	chr11:47350047	0.08740	0.1007
c.492C>T absent	rs3218719	chr11:47350027	0.02924	0.03450
c.506-12del present	rs11570050	chr11:47349933	0.6900	0.7171
c.706A>G absent	rs3729989	chr11:47348490	0.1178	0.1328
c.786C>T absent	rs11570058	chr11:47347892	0.1138	0.1290
c.3288G>A absent	rs1052373	chr11:47333236	0.3519	0.3187
Chance of achieving the same haplotype in 24 individuals by chance			=2.15*10^27^	=2.07*10^25^

**FIGURE 2:**
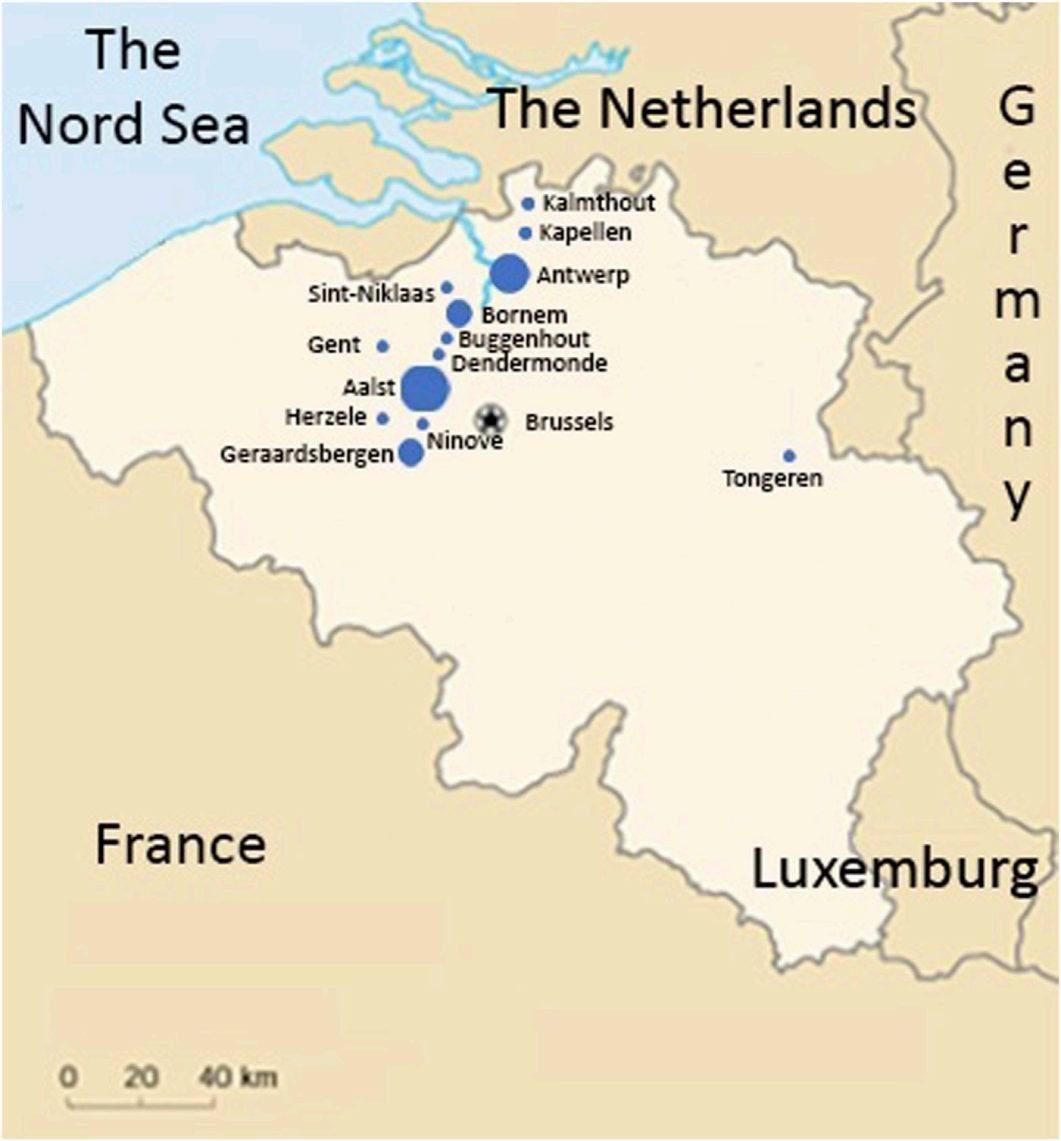
Overview of the location of the Belgian founders. The sizes of the dots represent the number of families identified in each city. All families live in a well-defined region between Brussels, Gent and Antwerp, centring around Aalst, except for one family identified in Tongeren in the East of Belgium.

The length of the common haplotype was assessed in all probands by 13 microsatellite markers spanning 26.4 Mb. A common haplotype of 1.19 Mb was identified in all 24 probands, with 19 of the probands sharing a 13.8 Mb haplotype (Figure 1). The founder event was estimated to have happened five generations ago. Taking into account that probands were on average 63 years old (ranging 50–87 years) and assuming that each generation spans circa 25 years (5*25 + 63), the founder event presumably took place 175–200 years ago (around the year 1830). All probands now live in a well-defined region within central Flanders, presumably the founder lived close to the region of Aalst (Figure 2).

### 3.5 Cardiac screening of relatives

Through cascade screening 59 first-degree relatives were genetically tested, of whom 37 (62.7%) were genotype positive (G+) and 22 (37.3%) genotype negative (G-). Screened family members were on average 38 ± 19 years old at the time of genetic testing. Differences between G+ and G- family members are displayed in Table 3.

**TABLE 3:** Overview of baseline characteristics and comparison between G- and G+ relatives.

	*MYBP3* del exon 23-26 family members		
	G- relative	G+ relative	*p*-value
	*N* = 22	*N* = 37	
Baseline characteristics			
Male gender (*n*, %)	7 (31.8)	14 (37.8)	0.641
Current age (y)	34.7 ± 14.7	41.6 ± 21.5	0.153
HCM phenotype (*n*, %)	1 (4.5)	19 (51.4)	**<0.001**
Myopathy (*n*, %)	0 (0)	1 (2.7)	1.000
IVS (mm)	10.1 ± 3.6	12.9 ± 4.1	0.073
PWT (mm)	9.5 ± 3.1	9.5 ± 2.1	0.945
Arterial hypertension (*n*, %)	3 (13.6)	13 (35.1)	0.200
Respiratory disease (*n*, %)	1 (4.5)	6 (16.2)	0.398

Del, deletion; G- relative, genotype negative relative; G+ relative, genotype positive relative; HCM, hypertrophic cardiomyopathy; IVS, intraventricular septum; PWT, posterior wall thickness; y, years. Bold values are significant differences.

Subsequent clinical assessment revealed an HCM phenotype in 19 (51.4%) G+ relatives, of whom only 5 (26.3% of newly identified HCM phenotypes) were symptomatic (NYHA class >1, angina, or syncope) at the time of genetic diagnosis (Table 1). G+ relatives with an HCM phenotype were older than those without an HCM phenotype (52.7 ± 21.3 vs. 30.6 ± 15.3; *p* < 0.001).

Characteristics of probands and G+ relatives are displayed in Table 1. Probands were older than G+ relatives (current age 63 ± 10 years vs. 42 ± 21 years; *p* < 0.001) and had more severe phenotypes with more clinical symptoms, non-sustained ventricular tachycardia (nsVT), more overt hypertrophy and left ventricular outflow tract obstruction (LVOTO) on echocardiography. The average interventricular septum thickness (IVS) was significantly higher in probands than G+/phenotype positive family members (20.6 ± 3.8 vs. 15.3 ± 3.5 mm; *p* < 0.001). Probands also presented with worse outcomes: more life-threatening arrhythmias, more heart failure hospitalizations, and a higher need for device therapy.

**FIGURE 3:**
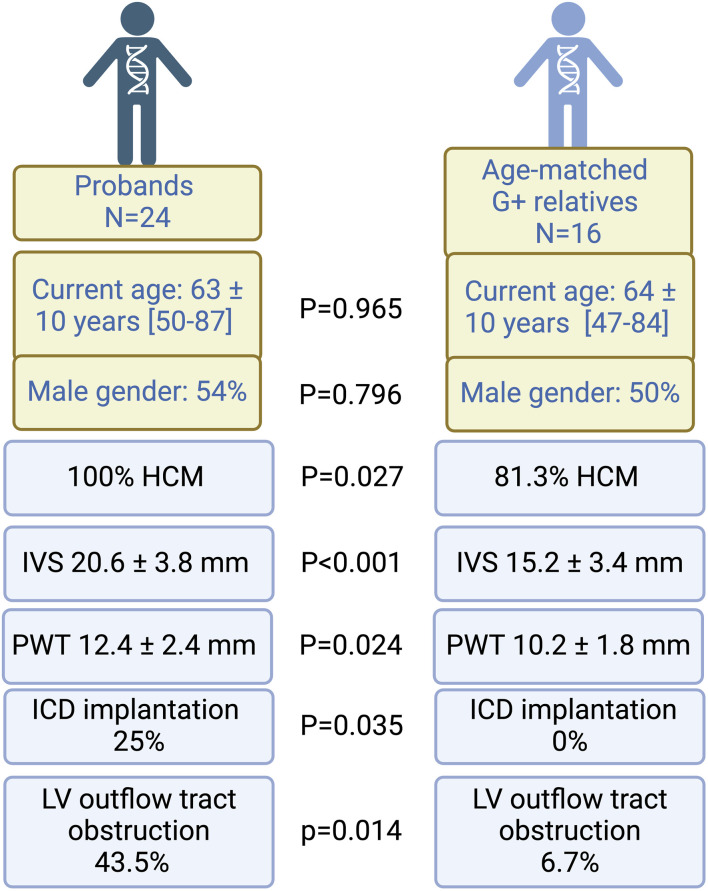
Comparison between probands and age-matched relatives. Probands have worse phenotypes, characterized by thicker left ventricles, a higher need for ICD implantation and more LV outflow tract obstruction. Proportions are displayed as percentages (%) of the comparison group (*n* = 24 for probands and *N* = 16 for G+ relatives). Echocardiography was performed in all individuals, however, a measurement of wall thickness was only available for 14 G+ relatives. G+, genotype positive; HCM, hypertrophic cardiomyopathy; ICD, implantable cardiac defibrillator; IVS, interventricular septum; LV, left ventricular; PWT, posterior wall thickness. Figure created with Biorender.

One might hypothesize that the differences between probands and G+ relatives is related to the age difference but when probands were compared to an age matched subgroup of G+ relatives (*n* = 16) we could still observe significant differences in hypertrophy (IVS 20.6 ± 3.8 vs. 15.2 ± 3.4; *p* ≤ 0.001) prevalence of device implantation (25.0% vs. 0%; *p* = 0.035) and prevalence of LVOTO (43.5% vs. 6.7%; *p* = 0.014) (Figure 3). Gender distribution was similar between probands and matched relatives. At the age of 50, a penetrance of 78.6% was observed, defined as the presence of HCM in 11 of 14 G+ relatives with age ≥50 years.

#### 3.5.1 Gender differences

Male G+ relatives more often had a HCM phenotype (78.6% vs. 34.8%; *p* = 0.010) and were more severely affected than females. They had thicker ventricles evidenced by an increased IVS thickness (15.5 ± 3.5 mm vs. 11.2 ± 3.6 mm; *p* = 0.003), increased posterior wall thickness (PWT, 11.0 ± 0.9 mm vs. 8.4 ± 2.0 mm; *p* < 0.001), a higher frequency of left atrial dilatation (58.3% of males vs. 9.5% of females; *p* = 0.002) and mitral valve regurgitation (75% of males vs. 20% of females; *p* = 0.002). Males also presented more frequently with repolarization disturbances on their rest ECG (58.3% of males vs. 21.1% of females; *p* = 0.035). However, when we corrected Left ventricular wall thickness (IVS and PWT) and Left atrial posterior-anterior diameter (LAAP) for body surface area (BSA), indexed measurements were similar between males and females (IVS 9.3 ± 2.2 mm/m^2^ vs. 9.2 ± 3.8 mm/m^2^; *p* = 0.441; LAAP 2.3 ± 0.3 cm/m^2^ vs. 2.2 ± 0.3 cm/m^2^; *p* = 0.560) and PWT was smaller in men compared to women (PWT 5.6 ± 0.7 vs. 6.5 ± 2.3 mm/m^2^; *p* = 0.049). In probands similar findings were present, with smaller IVS and PWT in men compared to women, when corrected for BSA (IVS: 10.6 ± 1.9 vs. 11.5 ± 3.6; *p* = 0.468 and PWT: 5.5 ± 0.8 vs. 7.5 ± 1.8 *p* = 0.02).

#### 3.5.2 Influence of environmental factors

G+ relatives with arterial hypertension (AHT) were more likely to have a HCM phenotype compared to G+ relatives without AHT (78.6% vs. 35%; *p* = 0.012). Smokers were not more likely to develop the HCM phenotype (57.1% of smokers vs. 51.9% of non-smoker had a HCM phenotype; *p* = 0.571). BMI tended to be higher in G+ relatives with a HCM phenotype (25.7 kg/m² ± 3.4 vs. 23.9 kg/m² ± 2.9; *p* = 0.201).

#### 3.5.3 Additional phenotypic characteristics

Unexpectedly, as *MYBPC3* encodes a cardiac specific isoform of MyBP-C, skeletal myopathy has been exceptionally described in association with *MYBPC3* variants (Tajsharghi et al., 2010; Song et al., 2023). In the current cohort, one G+ individual also had evidence of skeletal myopathy. No association with a respiratory phenotype (assessed as the presence of asthma or COPD) was seen in the current cohort.

### 3.6 Outcomes of all *MYBPC3* variant harbouring individuals

#### 3.6.1 Overall

Overall, 20.3% of all G+ patients have reached the composite cardiac outcome after a median follow-up of 5.5 years with an average age of 50 (±21) years (Table 1). Of carriers ≥50 years of age (n = 38), 11 (28.9%) had reached the composite endpoint.

#### 3.6.2 Effect of additional variant

A total of eleven (45.8%) probands carried an additional variant of uncertain significance (*n* = 10) or likely pathogenic variant (*n* = 1; *MIB1* c.1A>C) (Table 1). An overview of the additional variants in the proband population is given in Supplementary Table S1.

There were no differences in family history for HCM, SCD or disease severity in probands with an additional variant compared to the probands without.

In one family with eight del23-26 carriers, an additional *MYBPC3* VUS was identified in one individual (c.3763G>A; p.Ala1255Thr). In this family two patients had received a heart transplant and five patients experienced premature (sudden) cardiac death [at ages 24 (after HTx), 28, 50, 52 and 58 years]. Unfortunately, no DNA was available for three of the deceased patients, for two of the deceased patients (death at 50 and 52) presence of the founder variant could be confirmed based on the family tree. No individuals carrying only the VUS were identified. The patient who received a HTx and is still alive, is the family member who carries the additional *MYBPC3* VUS. Eleven G+-family members carried the additional variant identified in their respective probands. They were more likely to be symptomatic at time of diagnosis (57.1% vs. 38.5%; *p* = 0.021) and to reach the composite endpoint (27.3% vs. 4.2%; *p* = 0.082) than family members without the additional variant identified in the proband. No significant difference in degree of left ventricular hypertrophy was observed (IVS 13.1 ± 4.2 vs. 12.8 ± 4.1 mm; *p* = 0.847).

Of note, a HCM phenotype was identified In 1 G- family member carrying two variants of uncertain significance (*JUP* c.1096 C>G (p.Pro366Ala) and *CALR3* c.564delT (p.Gln189Serfs*8) (Identified with the full CM gene panel). Although *JUP* has so far only been linked to arrhythmogenic cardiomyopathy (Groeneweg et al., 2015) and the association of *CALR3* to HCM is disputed (Verhagen et al., 2018), we cannot exclude that these variants play a role in the presence and severity of HCM in this family. The *CALR3* variant was present in four additional family members, including the proband, the *JUP* variant was identified in two other family members, but was absent in the proband (Supplementary Material).

## 4 Discussion

The deletion of exon 23-26 of *MYBPC3* is a pathogenic Belgian founder mutation. Within the cohort of 61 mutation carriers we collected, the mutation leads to a composite cardiac outcome in >29% of individuals ≥50 years and shows a penetrance of >75% in G+ relatives above age 50.

In this work we described the presence of a larger intragenic deletion of four exons in *MYBPC3*. CNVs are a rare cause of cardiomyopathy and are present in up to 1.9% of HCM patients (Mates et al., 2020). Although copy number variants (CNVs) can be missed by standard genetic testing using Sanger or some next-generation sequencing techniques (Mates et al., 2020), multi-exon deletions can be detected based on NGS data, by comparing the normalized sequencing depth of exons between cases (Fromer et al., 2012). The *MYBPC3* exon 23-26 deletion in our population was identified based on NGS sequencing data using a read-depth strategy, showing it is feasible to pick these up. As data is already available and added time and cost is limited, we advise that such CNV analysis of the *MYBPC3* gene is routinely implemented in genetic analysis of HCM patients. We were able to pinpoint the exact breakpoints of this deletion using long-range PCR. To our knowledge, this is the first *MYBPC3* CNV described as a founder variant. We could identify a common haplotype of 1.19 Mb (including the 2 intragenic SNPs) in all 24 probands and a larger common haplotype of 13.8 Mb in 19 of the 24 assessed probands. This allowed us to estimate the founder event to have happened around the year 1830.

The variant was classified according the ACMG guidelines for CNVs (Riggs et al., 2020). Argument 2E “Both breakpoints are within the same gene” requires further assessment with additional standard ACMG criteria (PVS1or PM4) which were both present for the *MYBPC3* variant (Richards et al., 2015). However, these criteria are general and do not take gene-specific characteristics into account. Several re-assessments of these standard criteria have been proposed for specific genes, including *MYH7*, the second most important HCM-related gene (Kelly et al., 2018). For *MYBPC3* however, no gene-specific criteria have been proposed yet.

The deletion of exon 23-26 of *MYBPC3* is predicted to result in an unstable transcript due to the deletion of 589 nucleotides, which is expected to undergo nonsense-medited mRNA decay. Therefore, the effect of the variant is expected to be similar to that of other loss-of-function (haploinsufficient) variants in *MYBPC3*. Whereas pathogenic loss-of-function (haploinsufficient) variants are evenly distributed throughout the whole *MYBPC3* gene, pathogenic missense variants cluster in specific domains. Most pathogenic missense variants in *MYBPC3* result in absence or altering of the myosin binding site, although disruption of the titin binding site and phosphorylation sites can also result in HCM (Winegrad, 1999; Helms et al., 2020). It is likely that, opposed to a dominant-negative effect of missense mutations, nonsense-mediated decay and subsequent haploinsufficiency cause a HCM phenotype in this patient population (Rottbauer et al., 1997; Andersen et al., 2004; Frank-Hansen et al., 2008).

### 4.1 Phenotype-genotype correlations of the *MYBPC3* gene

A large phenotypic variability has been reported (Page et al., 2012; Helms et al., 2020) in *MYPBC3*-related HCM and this hinders the assignment of prognostic significance to specific disease-associated mutations or groups of mutations, complicating (genetic) counselling of family members.

Based on literature, the disease penetrance of *MYBPC3* variants was 58%–83% at age 50 years (Charron et al., 1998; Niimura et al., 1998; Maron et al., 2001), which resembles our founder population’s disease penetrance of 78.6% at age 50 (in family members). Average age of clinical onset for *MYBPC3*-harbouring individuals is around 40 years (Charron et al., 1998; Niimura et al., 1998; Van Driest et al., 2004; Sedaghat-Hamedani et al., 2018; Lorenzini et al., 2020b). In our *MYBPC3* founder population, age of clinical diagnosis was considerably later, with an average of 51 ± 17 years (probands and family members). Considering that the presence of HCM leads to a loss in quality of life, and HCM is often progressive, an earlier onset can be seen as a more severe phenotypic presentation when compared to late onset.

After the initial discovery of *MYBPC3*, several studies reported on a less severe phenotype in *MYBPC3* variants compared to other known HCM-related genes, especially *MYH7*. The degree of hypertrophy in our probands (IVS 20.6 mm) was similar to the described *MYBPC3* literature [20.6 (19-22)mm, current age not known (Sedaghat-Hamedani et al., 2018)]. However, when all G+ individuals were considered, IVS was lower (16.3 ± 5.5 mm). In a large meta-analysis of 31 studies with 6123 HCM patients, *MYBPC3* variant carriers (20% of all HCM patients) had less cardiac conduction disease, less ventricular arrythmia and less HTx (present in 15%, 20% and 0.6% of all MYBPC3 variant carriers respectively) compared to *MYH7* variant carriers (Sedaghat-Hamedani et al., 2018). We observed intraventricular conduction disturbances in 25.5% and ventricular arrhythmia in 25.5% of the G+ individuals in our cohort which was not significantly higher than the observation in the *MYBPC3* group described above (*p* = 0.1 for conduction disease and *p* = 0.47 for ventricular arrythmia). None of the patients who were followed in our cohort died during follow-up (5.5 years [1-22]), but three (5.2%) did experience a life-threatening arrhythmia.

A large study stratified *MYBPC3* families according to prognosis, from benign (no death or HTx before the age of 60 years in the family, including the proband) to intermediate (1 event in the family) and malignant (2 or more events in the family). Overall, 90% of families with a *MYBPC3* variant were stratified as having a benign or intermediate prognosis (Richard et al., 2003). According to this classification three families with the founder variant can be classified as malignant (12.5%), one family as intermediate (4.2%) and the other 20 families as benign (83.3%). This inter-familial variability indicates that even a single specific variant can be identified with a range of disease severities.

### 4.2 Benign phenotype of founder variants?

Most variants identified in *MYBPC3* are “private” variants, meaning that they have been identified in a single family. In a large cohort, up to 56% of variants could be considered private (Alfares et al., 2015). When a variant is identified in several families and a shared haplotype is characterized, it represents a founder variant. The proportion of founder variants in HCM patients differs from region to region with higher proportions in isolated populations. Most strikingly, in Iceland up to 58% of the complete HCM population has the same specific *MYBPC3* founder variant (*MYBPC3* c.927-2A>G), with an overall population prevalence of 0.36% (Adalsteinsdottir et al., 2014). Founder variants are suggested to be associated with a more benign phenotype, as they have withstood negative selection pressure (Jääskeläinen et al., 2004; Thompson and Day, 2017). In several populations, however, no difference in LV hypertrophy severity or prevalence of adverse events between founders and non-founders were observed (Christiaans et al., 2010a; Christiaans et al., 2010b; Ross et al., 2017; Sabater-Molina et al., 2017; van Velzen et al., 2017; Helms et al., 2020).

Our current cohort is characterized by a higher age of onset, but an overall high penetrance at the age of 50 years (78.6% of G+ family members, in comparison to an average penetrance of 50% at this age in genotype positive HCM (Lorenzini et al., 2020b). Despite this late onset, negative outcomes were frequent, suggesting a stronger contribution of this specific founder variant on the HCM phenotype penetrance.

Therefore, the presence of any (*MYBPC3*) genetic variant seems to be the most important predictor of phenotype and severity, rather than the nature of the exact *MYBPC3* variant itself. These *MYBPC3* founder variants could withstand negative selection pressure as overall HCM mortality is low and the mean age of HCM related mortality is around 68 years which is long after the age of reproduction (compared to 81 years for non-HCM related mortality, *p* = 0.02) (Adalsteinsdottir et al., 2014).

### 4.3 Difference in disease severity between probands and G+ relatives

In our study cohort, probands were significantly more affected than G+ relatives and some of these differences stayed significant after correction for age by comparison with an age-matched cohort. Probands had thicker left ventricles compared to age- and gender matched G+ family members with a HCM phenotype, had more outflow tract obstruction and worse outcomes. It should be noted however, that overall, probands were more extensively investigated than family members, with a higher proportion of probands undergoing cardiac MRI and longer follow-up periods.

A similar observation was present in other *MYBPC3* variant carrying populations, regardless of this being a founder variant or not. Overall, G+ relatives with a clinical HCM phenotype had less hypertrophy, less adverse remodeling and had lower risk of complications and mortality compared to probands (van Velzen et al., 2016; van Velzen et al., 2017). Age at time of genetic diagnosis was lower in G+ family members, but age at time of clinical HCM diagnosis was similar between probands and their family members (van Velzen et al., 2016; van Velzen et al., 2017). Likewise in our cohort, G+ family members were younger at the time of clinical diagnosis, which likely is explained by the active screening of individuals through cascade screening, whereas probands only present when symptoms are obvious, or complications have occurred.

### 4.4 Genetic and non-genetic influencing factors

Male sex is a significant predictor for the development of an HCM phenotype in G+ carriers who were phenotype negative at initial investigation. This is true for genotype positive HCM in general, *MYBPC3* carriers and *MYBPC3* founder carriers, including the founder population presented in the current work (Christiaans et al., 2010a; Page et al., 2012; Terauchi et al., 2015; Sabater-Molina et al., 2017; van Velzen et al., 2017; Lorenzini et al., 2020b). Additionally, female patients with HCM are diagnosed later than men (Adalsteinsdottir et al., 2014; Sabater-Molina et al., 2017; van Velzen et al., 2017; Lakdawala et al., 2021). However, women experience more heart failure symptoms than men, have more LV outflow tract obstruction and more frequently have a need for medical treatment (Adalsteinsdottir et al., 2014; Terauchi et al., 2015; Lakdawala et al., 2021). Several studies, including a large meta-analysis, on a total of 9,427 HCM patients (39.5% women), confirmed that during follow-up, women have a higher risk for the development of heart failure and all-cause mortality even after correction for differences present at baseline (Terauchi et al., 2015; Geske et al., 2017; Lorenzini et al., 2020a; Lakdawala et al., 2021; Trongtorsak et al., 2021).

Part of the explanation of a smaller portion of women in (*MYBPC3*-related) HCM could be the routine use of non-indexed measurements of wall thickness for the diagnosis of HCM. As men are on average larger and have thicker ventricle walls, they are more likely to fall above the threshold for hypertrophy (Lang et al., 2015). This is supported by the observation that differences in wall thickness were reversed after indexation for body surface area (van Velzen et al., 2017; Lakdawala et al., 2021). Gender-specific cut-offs, or the routine implementation of BSA indexed cut-offs is therefore warranted. This is further supported by the higher uptake of testing with a genetic panel in female HCM patients compared to male HCM patients (36% vs. 30% *p* < 0.001 and 51% vs. 43% *p* < 0.001) (Alfares et al., 2015; Lakdawala et al., 2021). All clinicians should be aware of this gender bias, as the delayed diagnosis in women seems to be related to worse outcomes.

In literature, the presence of an additional (likely) pathogenic variant in *MYBPC3* or another HCM-related gene seems to result in worse phenotypes. Homozygosity or compound heterozygosity is present in 0.6%–7.5% of HCM patients and is associated with earlier onset and more severe phenotype (Van Driest et al., 2004; Ingles et al., 2005; Girolami et al., 2010; Marziliano et al., 2012; Page et al., 2012; Teirlinck et al., 2012; Alfares et al., 2015; Lorenzini et al., 2020b). The presence of a VUS next to a (likely) pathogenic variant has been observed in 7.4%–9.8% of HCM patients, but the effect of an additional VUS on the phenotype and disease severity has not been well defined (Alfares et al., 2015; Lakdawala et al., 2021).

In our cohort, 45.8% of probands carried one (or more) additional variants in a gene associated with cardiomyopathy, most of these were VUS, but in one family an additional likely pathogenic/pathogenic *MIB1* c.1A>C variant (4.2%) was present. We could not observe any clear differences between probands with or without an additional variant. It should be taken into account that VUS encompasses a broad range of variants, and some VUS might be leaning more to likely pathogenic (hot) whereas others are more likely to be benign after all.

As family members only underwent testing of the *MYBPC3* deletion, the presence of additional variants within the 59 CM genes, functioning as genetic modifiers cannot be excluded. As the probands were the most severely affected individuals, we expect genetic modifiers that worsen the phenotype to be, preferentially, identified in these probands. We showed that the G+ family members carrying the additional variant of the proband presented with a more severe phenotype than G+ family members without the additional variant, suggesting that some of the additional variants could act as aggravating modifiers having a synergistic effect on the MYBPC3 founder variant. Identification in more individuals and experiments investigating the actual functional effect of these variants should be performed to prove this hypothesis. Concerning potential protective genetic modifiers that lead to a less severe phenotype, we would expect to detect them in the family members, but we did not perform the full panel screening in these individuals and as such have no data to investigate this. We did not collect genetic data outside of the 59 CM genes, so cannot investigate protective or aggravating modifiers in other parts of the genome. Genome-wide linkage analysis based on short tandem repeat markers or genome-wide associations studies (GWAS) using common SNPs can aid to identify genetic modifiers. However, large families for linkage analysis and large cohorts of patients for GWAS are needed. Additionally, a large number of SNPs or markers need to be assessed, rendering this time- and cost consuming (Ott et al., 2015).

Overall, large differences in penetrance and disease severity are present in patients with the same genotype. Differences in the genetic background can be part of the explanation. Besides compound heterozygosity, more frequent genetic variation based on single nucleotide polymorphisms may play a role. Indeed, it has been shown that a polygenic risk score for HCM could stratify the HCM risk in family members of G+ probands (Zheng et al., 2023). Furthermore, this score aided the prediction of adverse outcomes in HCM patients (Zheng et al., 2023). However, the use of a polygenic risk score requires additional sequencing in all individuals, requiring extra time- and cost investment, which might not be feasible for all patients at the moment. For the current cohort, the data on the SNPs included in the risk score were not available to further stratify HCM risk.

Additionally, environmental factors may play a role. In our cohort G+ relatives with AHT were more likely to present with a HCM phenotype, whereas smoking or BMI did not significantly influenced disease penetrance. Interestingly, a marked difference in disease severity has been observed in three pairs of monogenic twins with a *MYBPC3* (c.787G>T; p.Gly263ter) founder variant (Rodríguez Junquera et al., 2022). As they are genetically identical, these differences cannot be explained by genetic background. The authors could not find any clear differences in known environmental risk factors for HCM such as AHT or physical activities (including pregnancy) (Rodríguez Junquera et al., 2022). Other, currently undiscovered, influencing factors (e.g., epigenetics) are therefore likely at play.

## 5 Limitations and future perspectives

A deletion of exon 23-26 of the *MYBPC3* gene has been reported before in one patient based in the UK and another patient based in the Netherlands, both were HCM patients (Nannenberg et al., 2011; Walsh et al., 2017). Although the exact breaking points of the deletion in these patients has not been identified, they most likely represent the same variant. Therefore, the fact that the haplotype in these two patients was not assessed and these individuals were not included in the current cohort is a limitation of the study.

Even though 24 probands with the *MYBPC3* del exon 23-26 variant have been identified, the overall sample size is limited. For follow-up of patients with limited disease, not all clinical tests (24 rhythm monitoring and cardiac MRI) were performed, further limiting our sample size and statistical power. Additionally, median follow-up is 5.5 years (7.0 [1-22] in probands, 3.5 [1-20] in G+ family members and 5.7 [1-20] in G-family members). Further follow-up of the current cohort is needed to assess long-term health outcomes in variant carriers and disease penetrance, especially in young G+ family members in whom a phenotype is currently absent.

## 6 Conclusion

We describe a Belgian founder variant, the deletion of exon 23-26 in *MYBPC3* associated with HCM. We identified 24 probands and subsequent cascade screening of 59 relatives identified 37 additional carriers. A common intragenic haplotype was confirmed in all 24 probands and a haplotype of 13.8 Mb in 19 probands could date the founder event to around 1830. This founder variant seems to be associated with a later onset (average age of clinical diagnosis around 50 years), nonetheless, above the age of 50 years a penetrance of 78.6% was observed. Despite this late onset, conduction disorders, outflow tract obstruction and ventricular arrhythmias were frequent, and the variant is associated with a high prevalence (20.3% overall) of adverse outcomes (HTx, life threatening arrhythmia, heart failure hospitalization or the need for septal ablation).

## Data Availability

The data presented in the study are deposited in the Clinvar repository https://www.ncbi.nlm.nih.gov/clinvar/, accession number VCV000525134.1.

## Abbreviations

AHT, arterial hypertension; CNV, copy number variant; G+/G-, genotype positive/negative; HCM, hypertrophic cardiomyopathy; HTx, heart transplantation; ICD, implantable cardiac defibrillator; IVS, interventricular septum; LAAP, left atrial anterior-posterior diameter; LV, left ventricle/Left ventricular; *MYBPC3*, Coding gene for myosin binding protein C; *MYH7*, Coding gene for myosin heavy chain 7 protein; PWT, posterior wall thickness; SCD, sudden cardiac death; Y, year(s).
