# Supplementary material for: Phenotypic spectrum of the first Belgian MYBPC3 founder: a large multi-exon deletion with a varying phenotype
Source: Front Genet. 2024 May 21;15:1392527. doi: 10.3389/fgene.2024.1392527 (PMC11148247; doi:10.3389/fgene.2024.1392527)
Supplement: Supplementary file 1 [file Table1.docx]

**Supplementary data**

**Supplementary Table 1 Overview of genes included in panel.**

| **CM 59 gene panel** |
| --- |
| *ABCC9* (ENST00000261200; ENST00000261201 exon 38), *ACTC1* (ENST00000290378), *ACTN2* (ENST00000542672; ENST00000366578 exon 8), *ALPK3* (ENST00000258888), *ANKRD1* (ENST00000371697), *BAG3* (ENST00000369085), *CALR3* (ENST00000269881), *CAV3* (ENST00000343849), *CRYAB* (ENST00000526180; ENST00000524660 exon1; ENST00000533971 for exon 2), *CSRP3* (ENST00000265968), *CTNNA2* (ENST00000433211), *DES* (ENST00000373960), *DSC2* (ENST00000280904; ENST00000251081 exon 16), *DSG2* (ENST00000261590), *DSP* (ENST00000379802), *EMD* (*STA*) (ENST00000369842), *FHL1* (ENST00000394155; ENST00000370683 exon 1 and 6), *FLH2* (ENST00000409177); *FLNC* (ENST00000325888); *GLA* (ENST00000218516), *JUP* (ENST00000393931), *JPH2* (ENST00000372980), *LAMA4* (ENST00000230538), *LAMP2* (ENST00000371335; ENST00000434600 exon 9; ENST00000200639 exon 9), *LDB3* (ENST00000429277; ENST00000372066 exon 4; ENST00000372056 exon 8; ENST00000361373 exon 7), *LMNA* (ENST00000368300; ENST00000368297 exon 2 and 11), *MIB1* (ENST00000261537), *MYBPC3* (ENST00000545968), *MYH6* (ENST00000405093), *MYH7* (ENST00000355349), *MYL2* (ENST00000228841), *MYL3* (ENST00000292327), *MYOZ2* (ENST00000307128), *MYPN* (ENST00000358913), *NEBL* (ENST00000377122; ENST00000417816 exon 1,2,3 and 4), *NEXN* (ENST00000334785), *PKP2* (ENST00000070846), *PLN* (ENST00000357525), *PPA* (ENST00000341595), *PRDM16* (ENST00000270722), *PRKAG2* (ENST00000287878), *RAF1* (ENST00000442415); *RBM20* (ENST00000369519), *RY*R2 (ENST00000366574), *SCN5A* (ENST00000333535; ENST00000413689 exon 6), *SDHA* (ENST00000264932); *SGCD* (ENST00000337851, ENST00000517913), *SYNE1* (ENST00000367255); *TAZ* (ENST00000601016), *TCAP* (ENST00000309889), *TGFB3* (ENST00000238682), *TMEM43* (ENST00000306077), *TNNC1* (ENST00000232975), *TNNI3* (ENST00000344887), *TNNT2* (ENST00000236918), *TPM1* (ENST00000403994; ENST00000334895 exon 1 and 8; ENST00000559397 exon 2; ENST00000358278 exon 6; ENST00000288398 exon 9; ENST00000559556 exon 9), *TTN* (ENST00000589042; ENST00000360870 exon 46), *TTR* (ENST00000237014), *VCL* (ENST00000211998). |

***Supplementary Table 2: Overview of additional variants identified in probands and their families.*** Genes previously associated with HCM are underlined. Definite HCM genes(Ingles et al., 2019) are in bold.

| **Proband** | **Additional variant identified** |
| --- | --- |
| **1** | / |
| **2** | / (an additional ***MYBPC3*** c.3763G>A; p.Ala1255Thr (VUS) in trans. |
| **3** | *CALR* c.564delT; p.Gln189Serfs*8 (VUS) |
| **4** | / |
| **5** | / |
| **6** | *FLNC* c.7652A>G; p.(Asp2551Gly) (VUS) |
| **7** | / |
| **8** | *MYH6* c.4772A>G; p.(Asn1591Ser) (VUS)  *JPH2* c.1756G>A; p.(Glu586Lys) (VUS) |
| **9** | *DSP* c.2622C>G; p.(Ile874Met) (VUS) |
| **10** | / |
| **11** | *DSP* c.5570A>C; p.(Lys1857Thr) (VUS)  *PRDM16* c.598A>G; p.(Ile200Val) (VUS) |
| **12** | / |
| **13** | / |
| **14** | / |
| **15** | ***ACTC1*** c.455-6G>A variant in intron 3 (VUS)  *DES* c.216C>A; p.(Ser72Arg) (VUS) |
| **16** | / |
| **17** | / |
| **18** | / |
| **19** | *RYR2* c.5154G>C; p.(Arg1718Ser) (VUS) |
| **20** | *DSP* c.3764G>A; p.(Arg1255Lys) (VUS) |
| **21** | *MIB1* c.1A>C; p.(?) (likely pathogenic) |
| **22** | / |
| **23** | ***MYH7*** c.1681G>A; p.(Ala561Thr) (VUS) |
| **24** | *FLNC* c.7652A>G; p.(Asp2551Gly) (VUS)  *FLNC* c.3967G>A; p.(Val1323Met) (VUS)  *RYR2* c.5638G>A; p.(Glu1880Lys) (VUS) |
