# Supplementary material for: Phenotypic spectrum of the first Belgian MYBPC3 founder: a large multi-exon deletion with a varying phenotype
Source: Front Genet. 2024 May 21;15:1392527. doi: 10.3389/fgene.2024.1392527 (PMC11148247; doi:10.3389/fgene.2024.1392527)
Supplement: Supplementary file 2 [file DataSheet1.PDF]

# Family trees

## Family 1

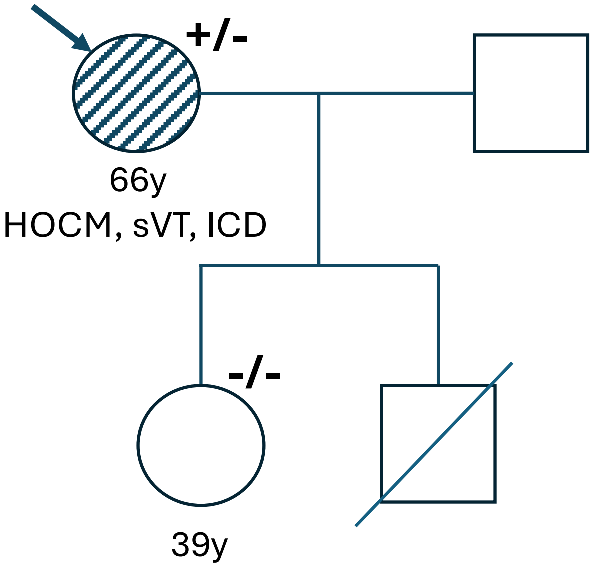

## Family 2

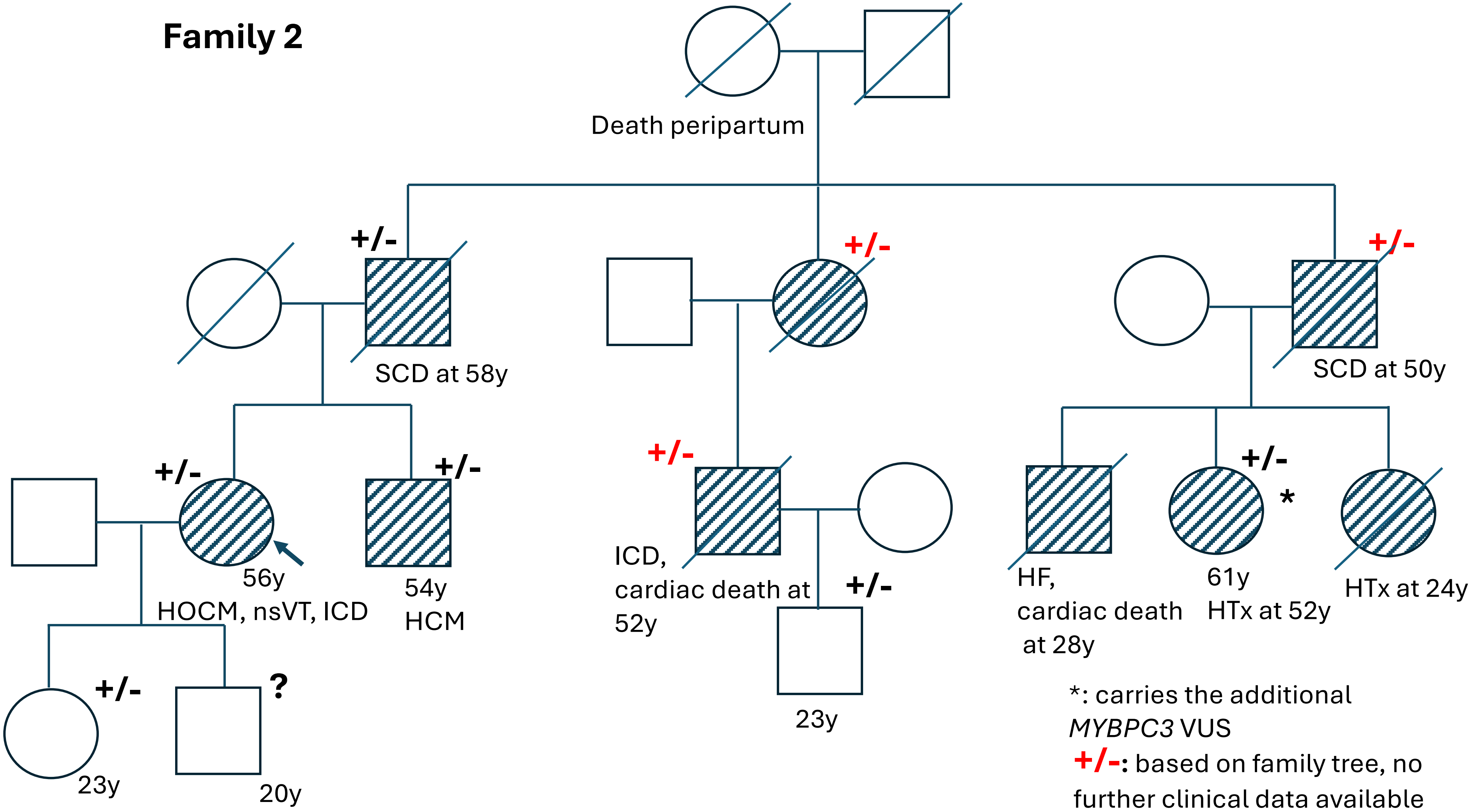
Death peripartum
+/-
SCD at 58y
+/-
+/-
SCD at 50y
+/-
56y
HOCM, nsVT, ICD
+/-
54y
HCM
+/-
ICD,
cardiac death at
52y
+/-
23y
+/-
*
HF,
cardiac death
at 28y
61y
HTx at 52y
HTx at 24y
+/-
23y
?
20y
*: carries the additional
*MYBPC3* VUS
+/-: based on family tree, no
further clinical data available

**Family 3**

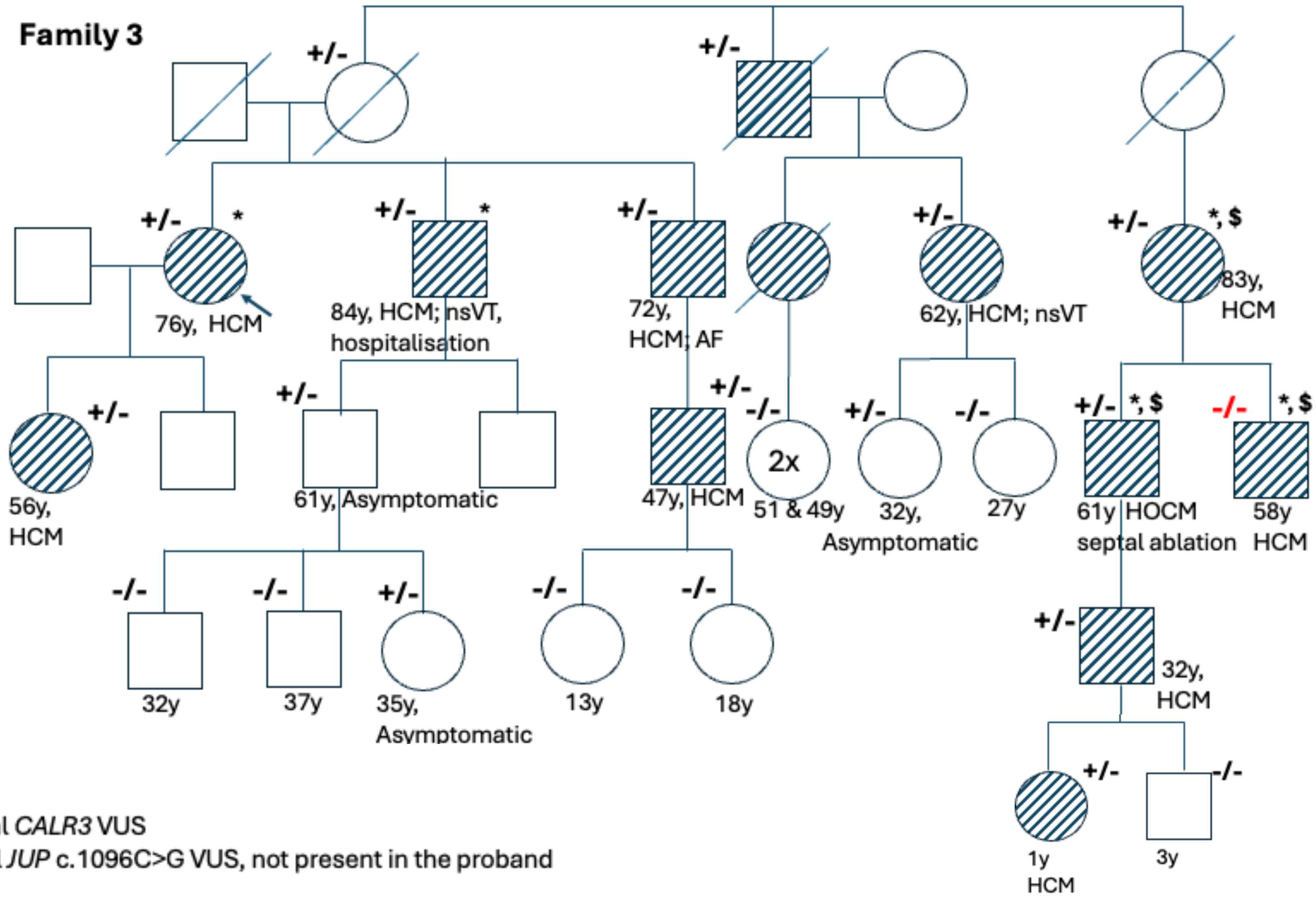


*: carries the additional *CALR3* VUS

$: carries an additional *JUP* c.1096C>G VUS, not present in the proband

## Family 4

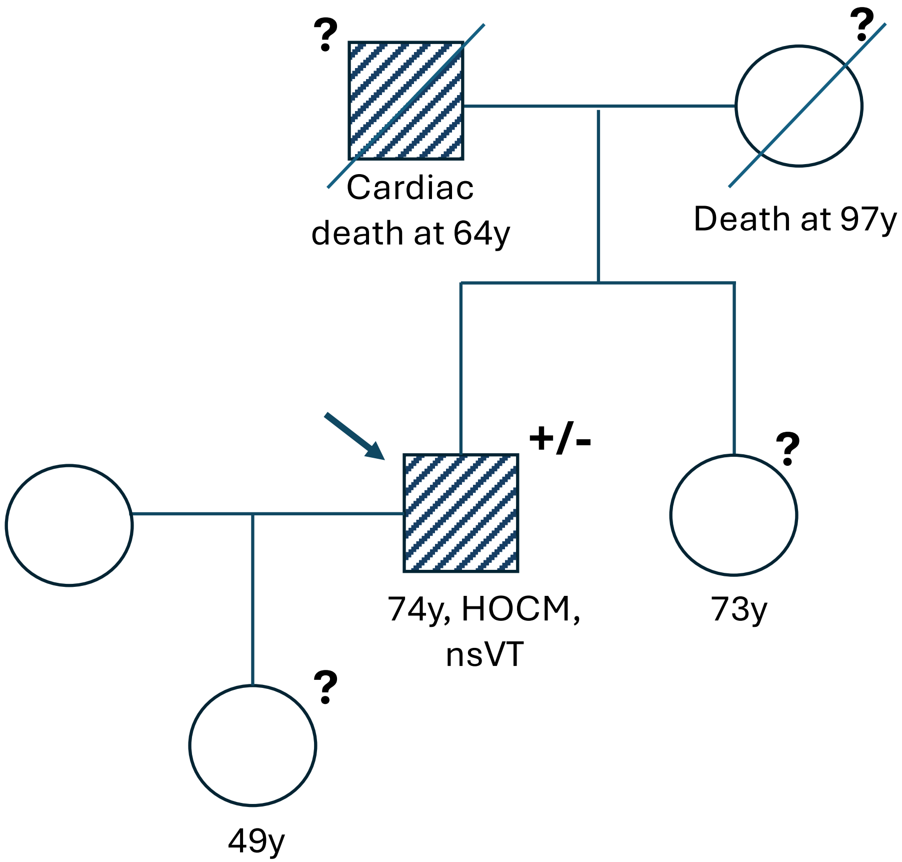

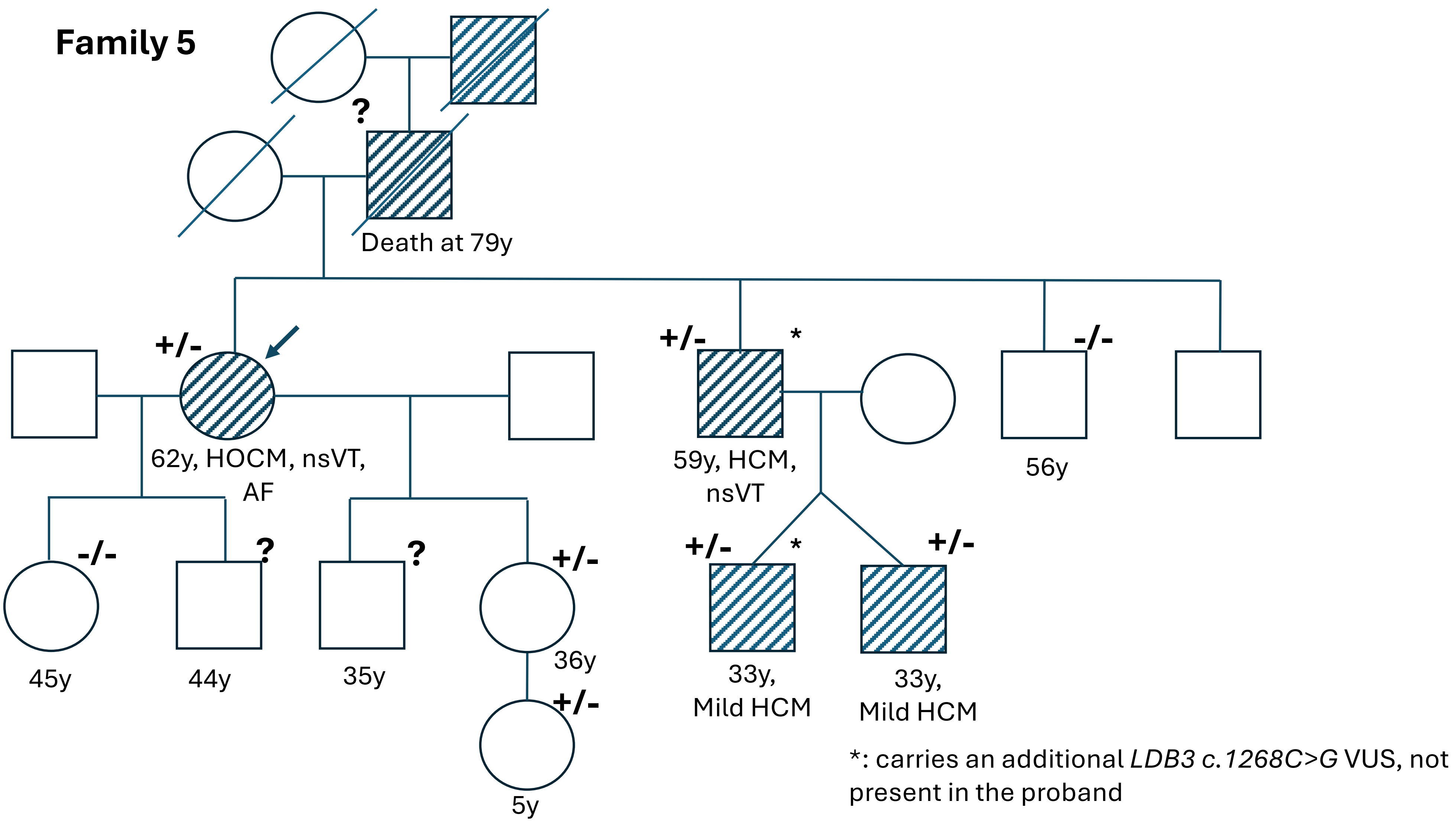
Family 5
?
Death at 79y
+/-
62y, HOCM, nsVT, AF
+/-
*
59y, HCM, nsVT
-/-
56y
-/-
45y
?
44y
?
35y
+/-
36y
+/-
5y
+/-
*
33y, Mild HCM
+/-
33y, Mild HCM
*: carries an additional *LDB3 c.1268C>G* VUS, not present in the proband

**Family 6**

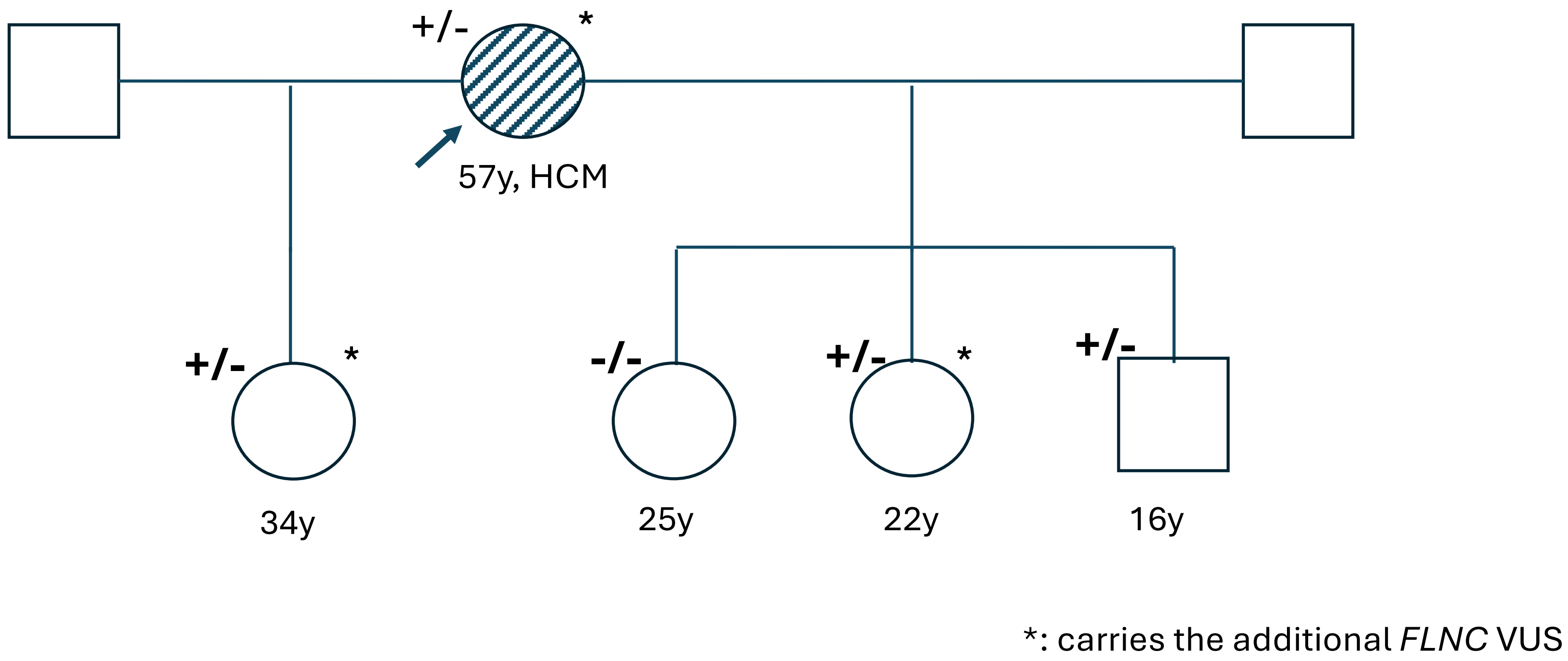

## Family 7

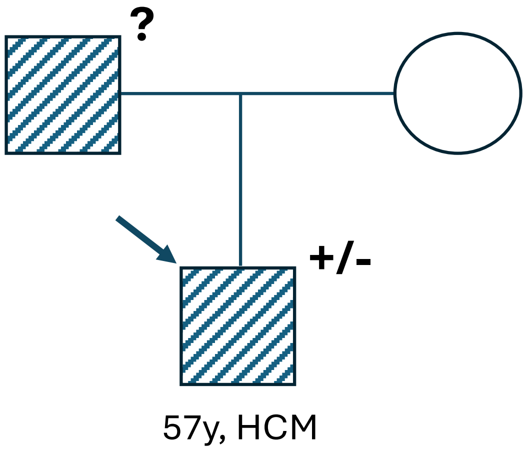

## Family 8

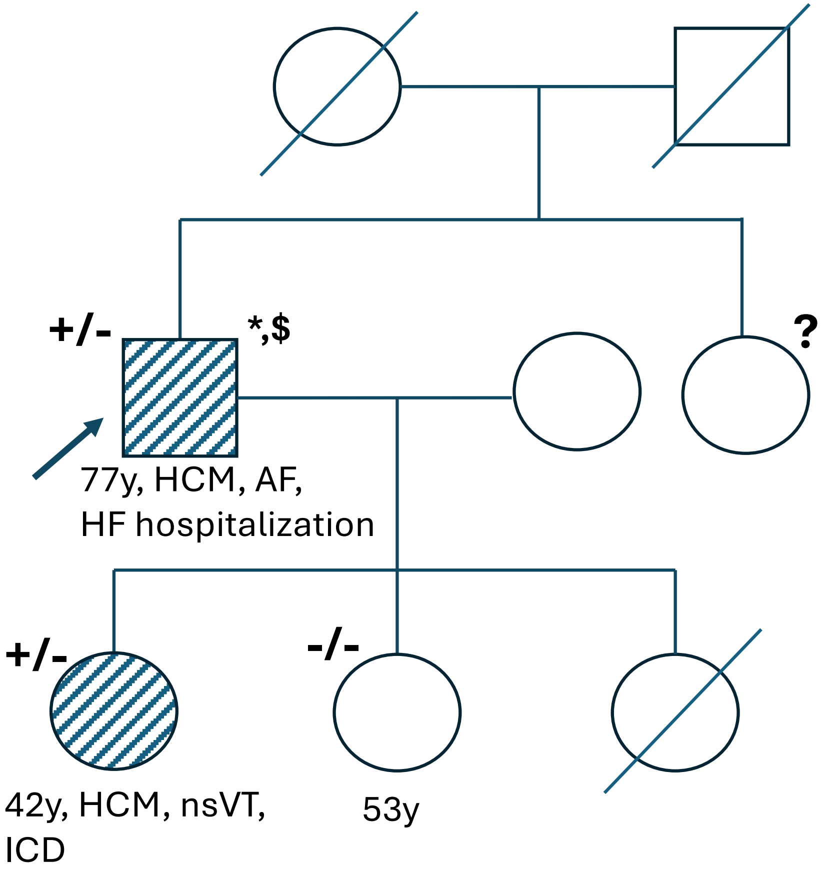


*: carries the additional *MYH6* VUS
$: carries the additional *JPH2* VUS

## Family 9

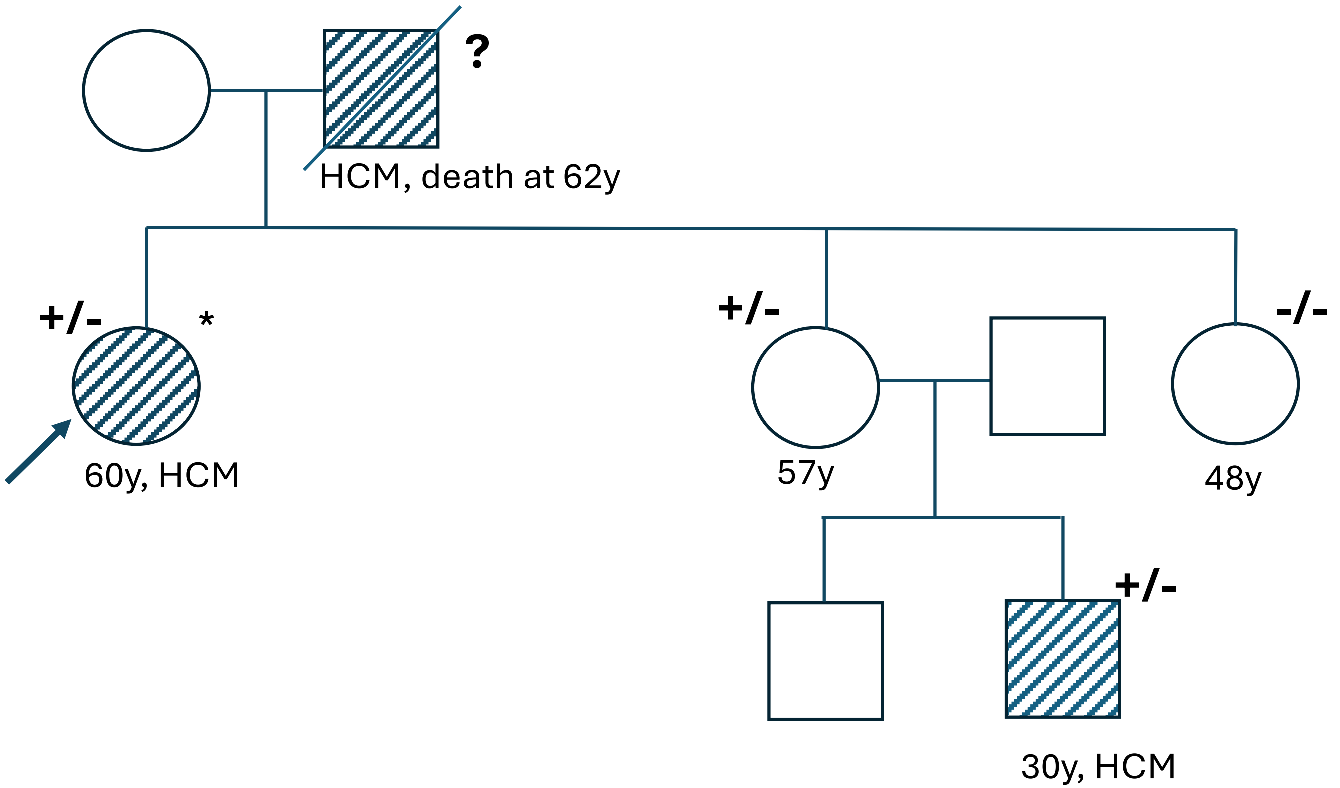


*: carries the additional *DSP* VUS

**Family 10**

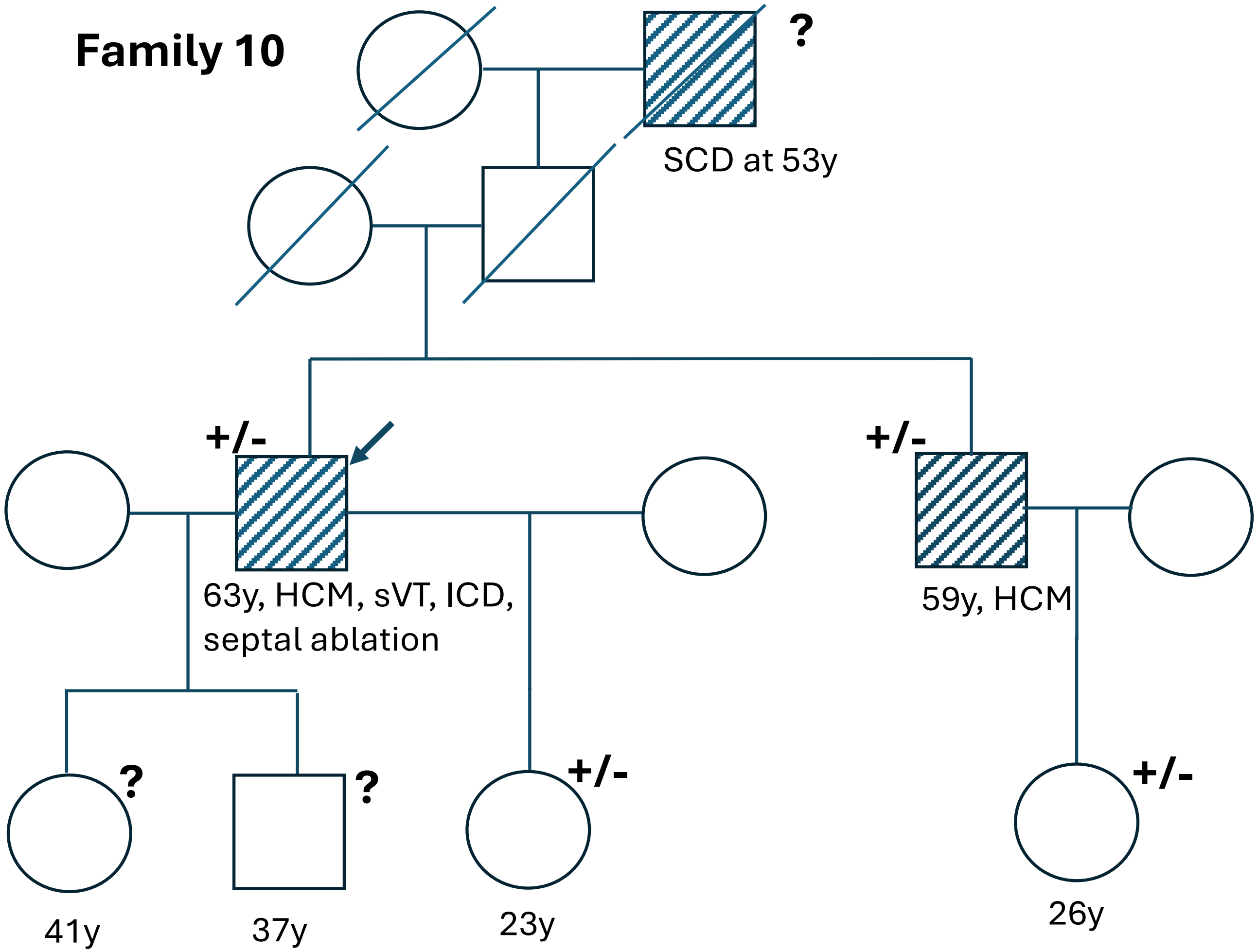
?
SCD at 53y
+/-
+/-
63y, HCM, sVT, ICD, septal ablation
59y, HCM
?
?
+/-
+/-
41y
37y
23y
26y

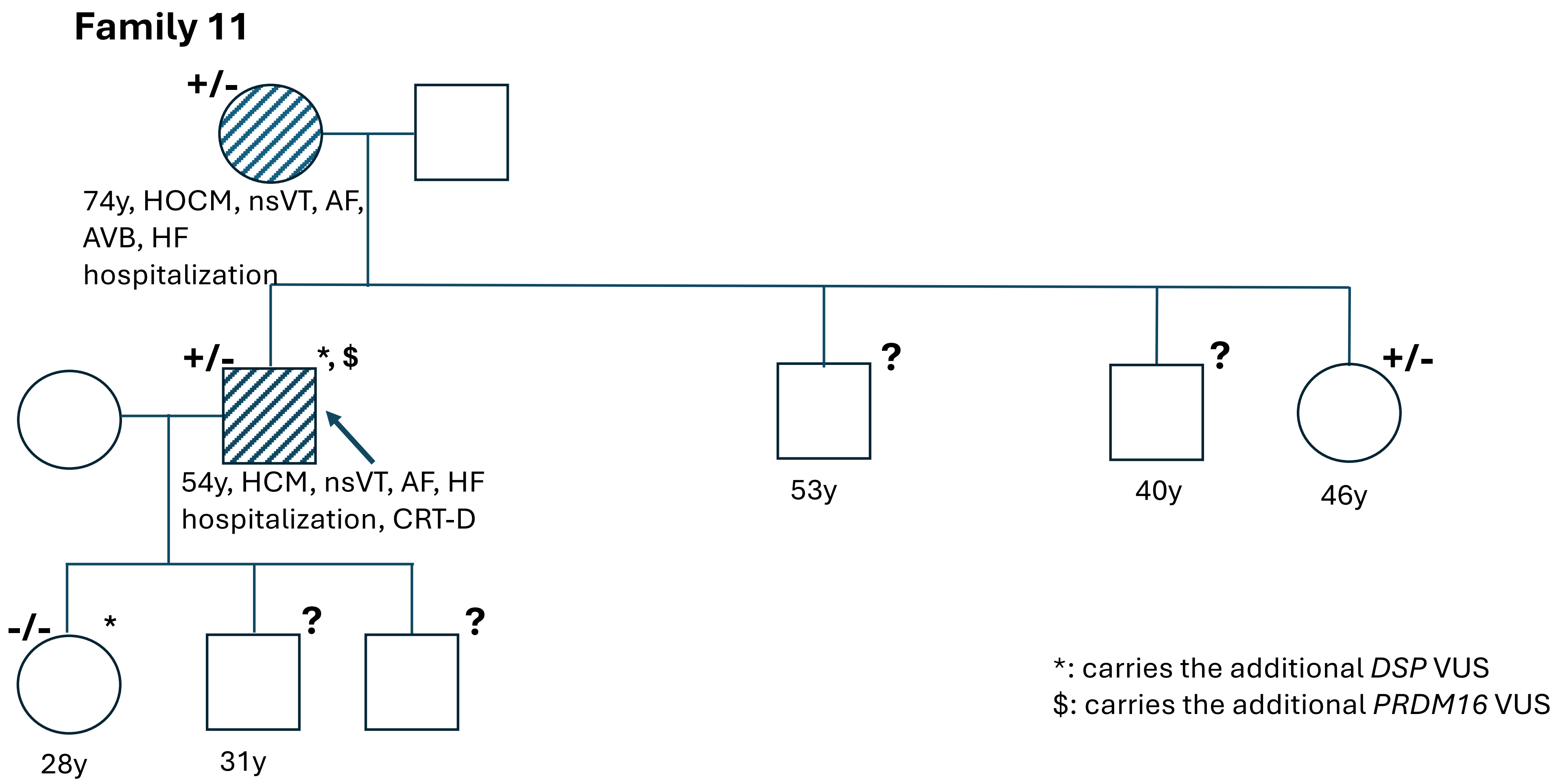
Family 11
+/-
74y, HOCM, nsVT, AF, AVB, HF hospitalization
+/-
*, $
54y, HCM, nsVT, AF, HF hospitalization, CRT-D
?
53y
?
40y
+/-
46y
-/-
*
28y
?
31y
?
*: carries the additional *DSP* VUS
$: carries the additional *PRDM16* VUS

## Family 12

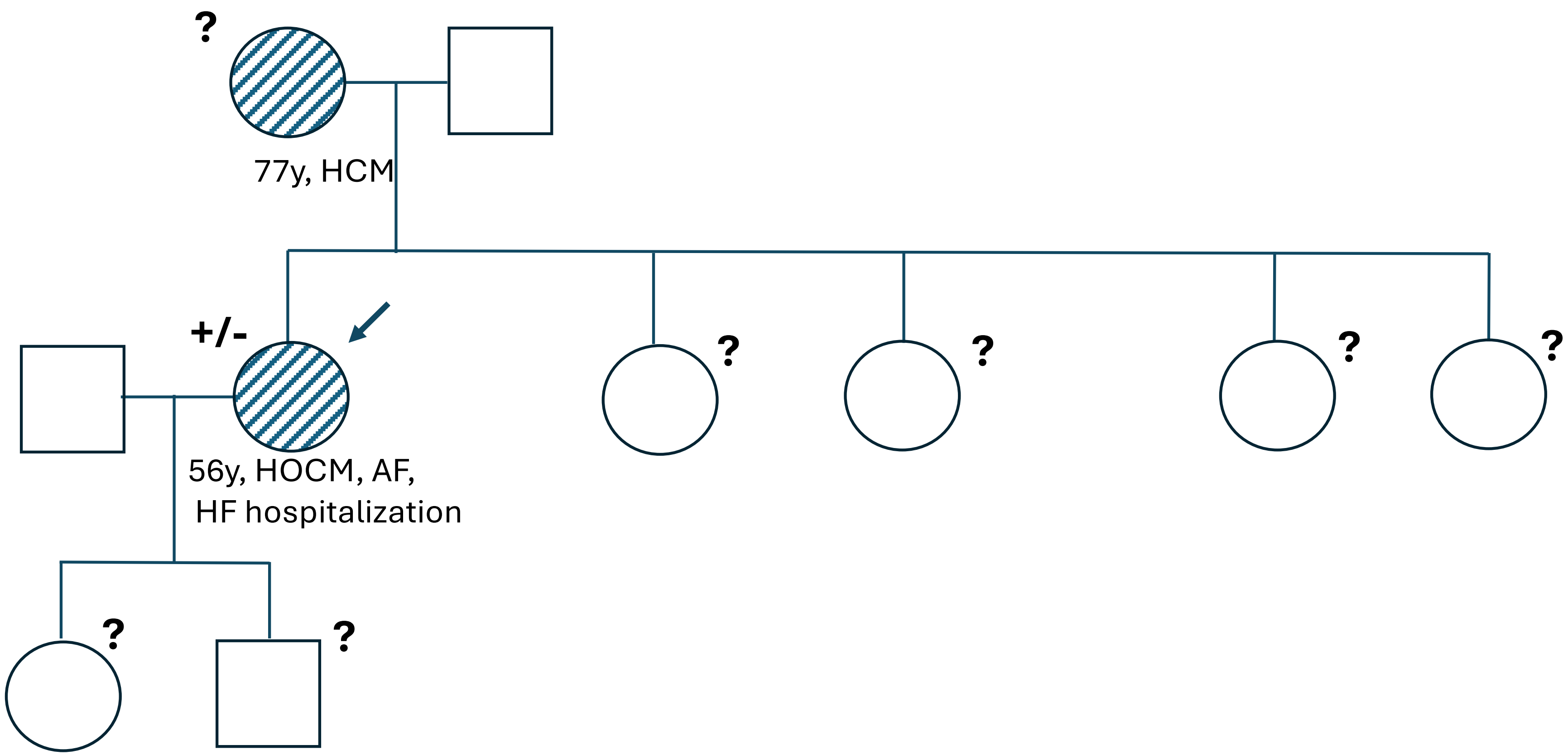

## Family 13

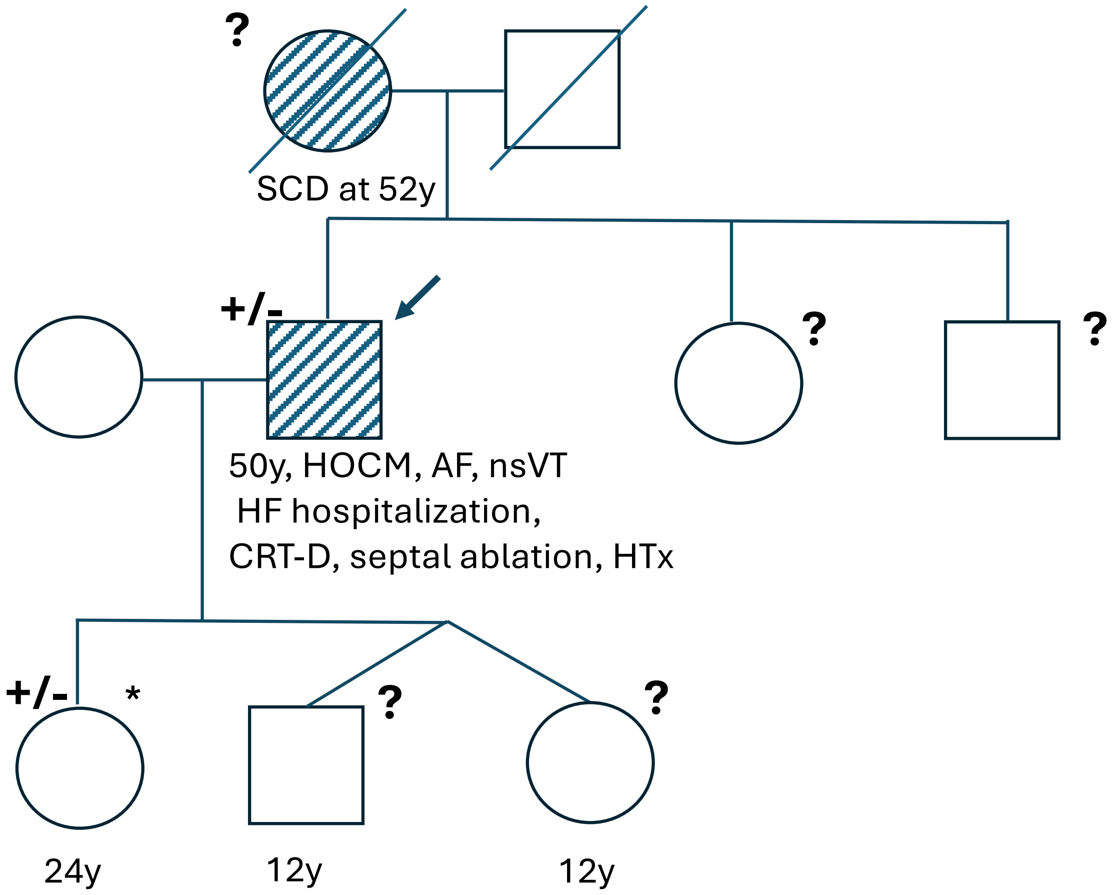


*: carries an additional *JUP* c.1130G>A VUS, not present in the proband

## Family 14

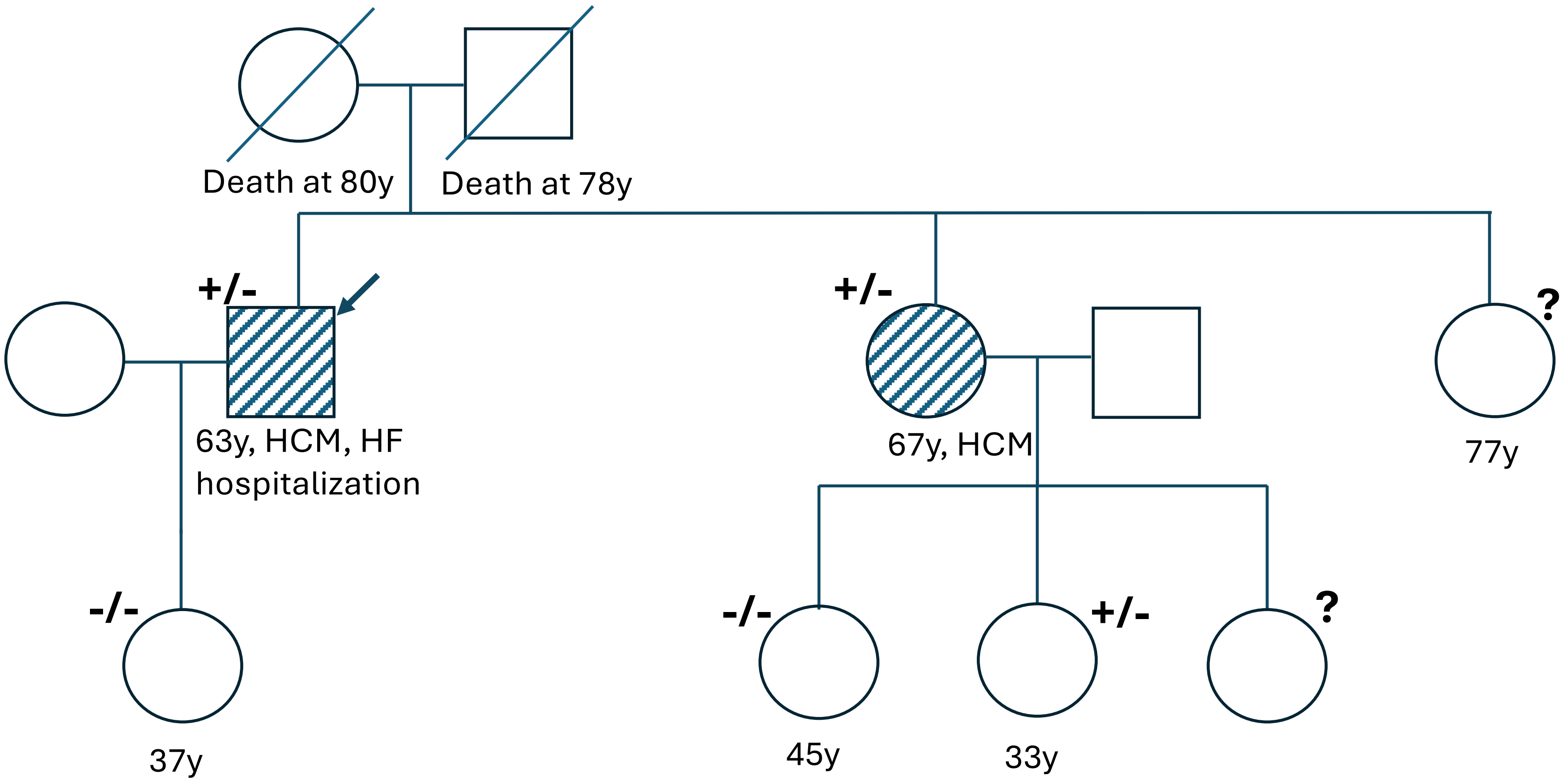

Death at 80y
Death at 78y
+/-
63y, HCM, HF hospitalization
+/-
67y, HCM
?
77y
-/-
37y
-/-
45y
+/-
33y
?

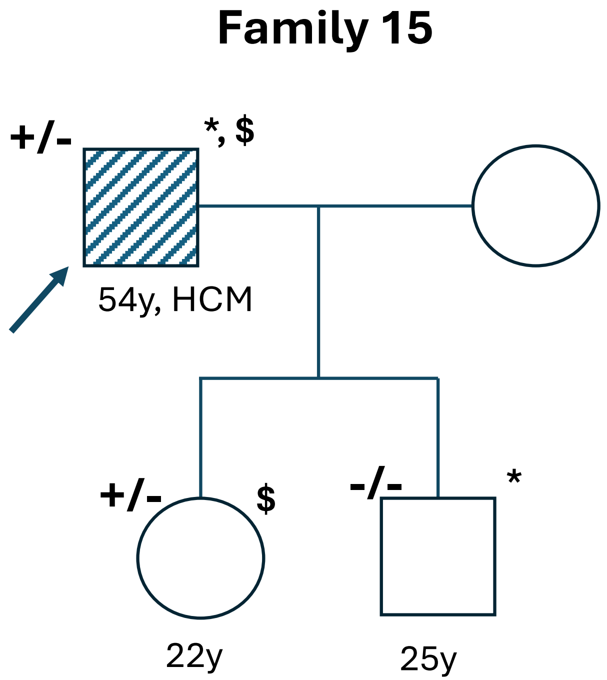


*: carries the additional *ACTC1* VUS
$: carries the additional *DES* VUS

## Family 16

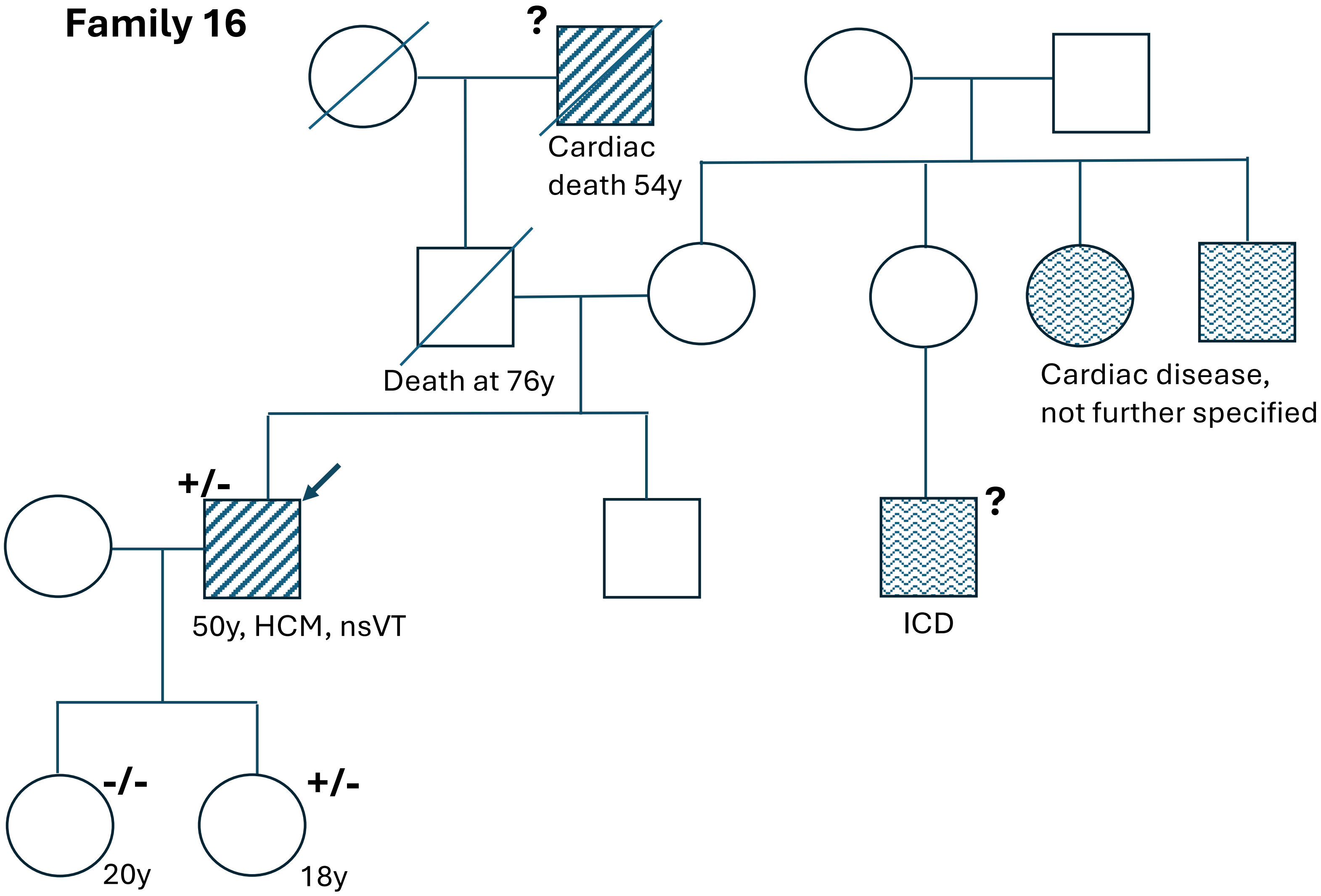

?
Cardiac death 54y
Death at 76y
Cardiac disease, not further specified
+/-
50y, HCM, nsVT
?
ICD
-/-
20y
+/-
18y

## Family 17

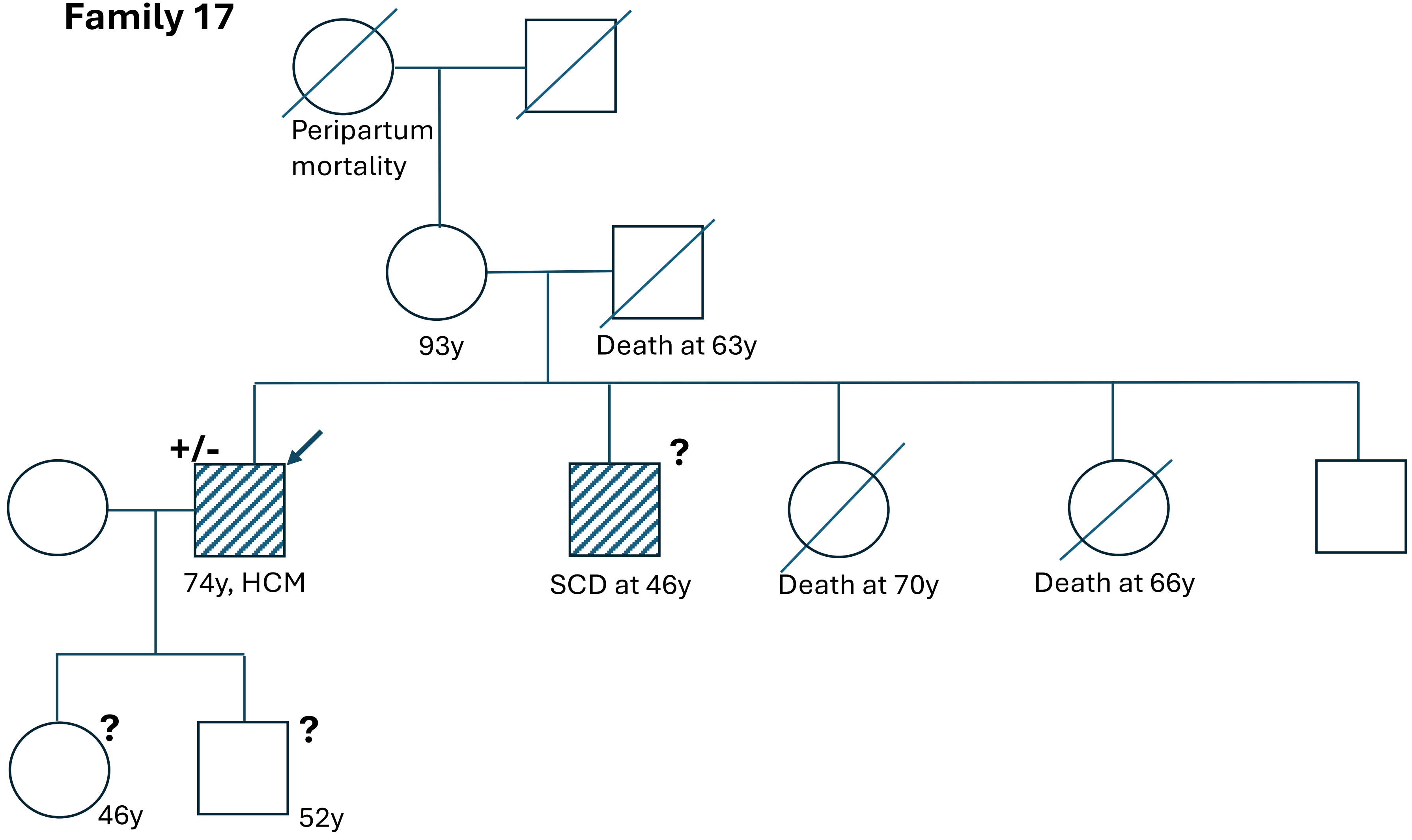
Peripartum
mortality
93y
Death at 63y
+/-
74y, HCM
?
SCD at 46y
Death at 70y
Death at 66y
?
46y
?
52y

## Family 18

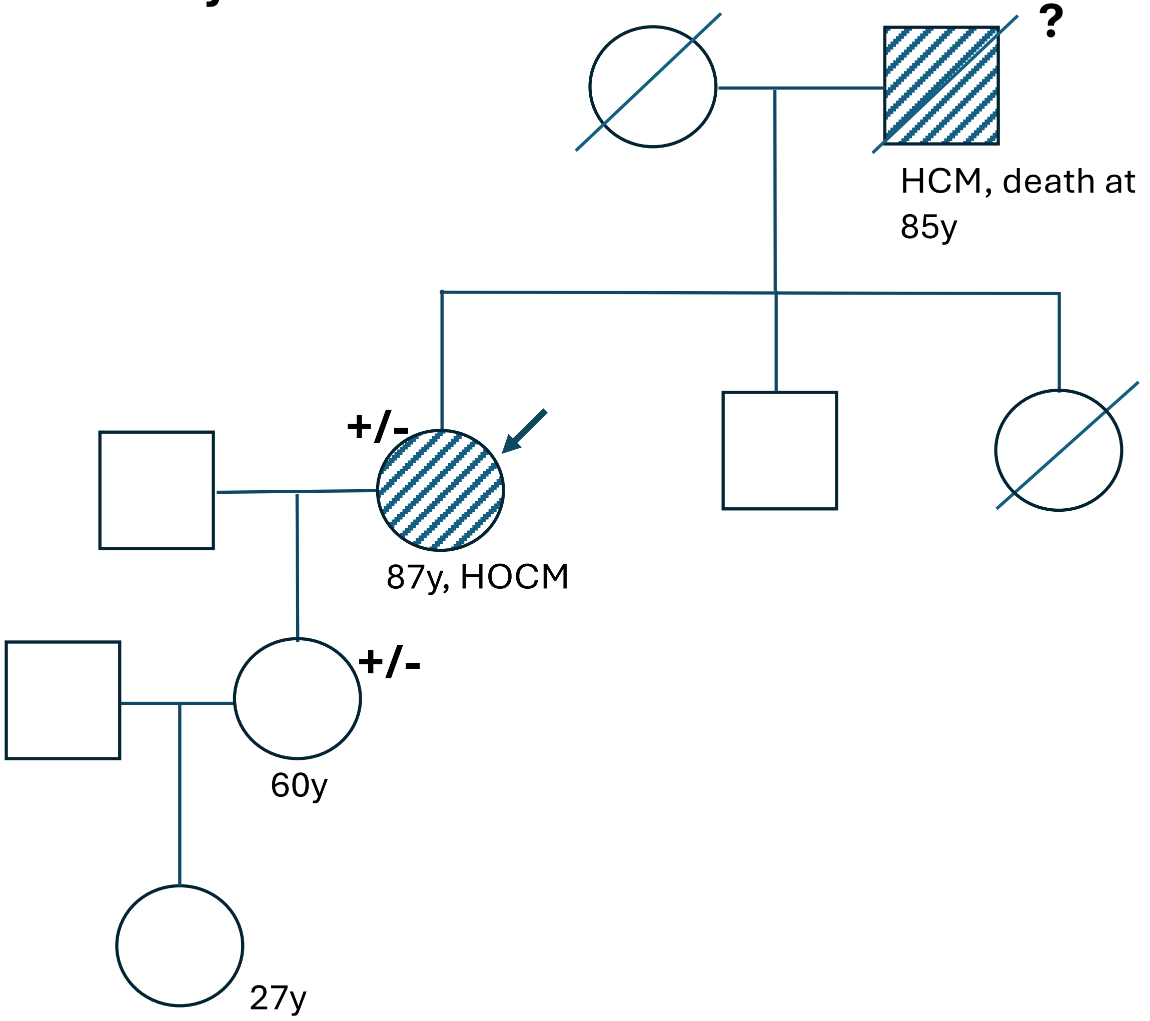

## Family 19

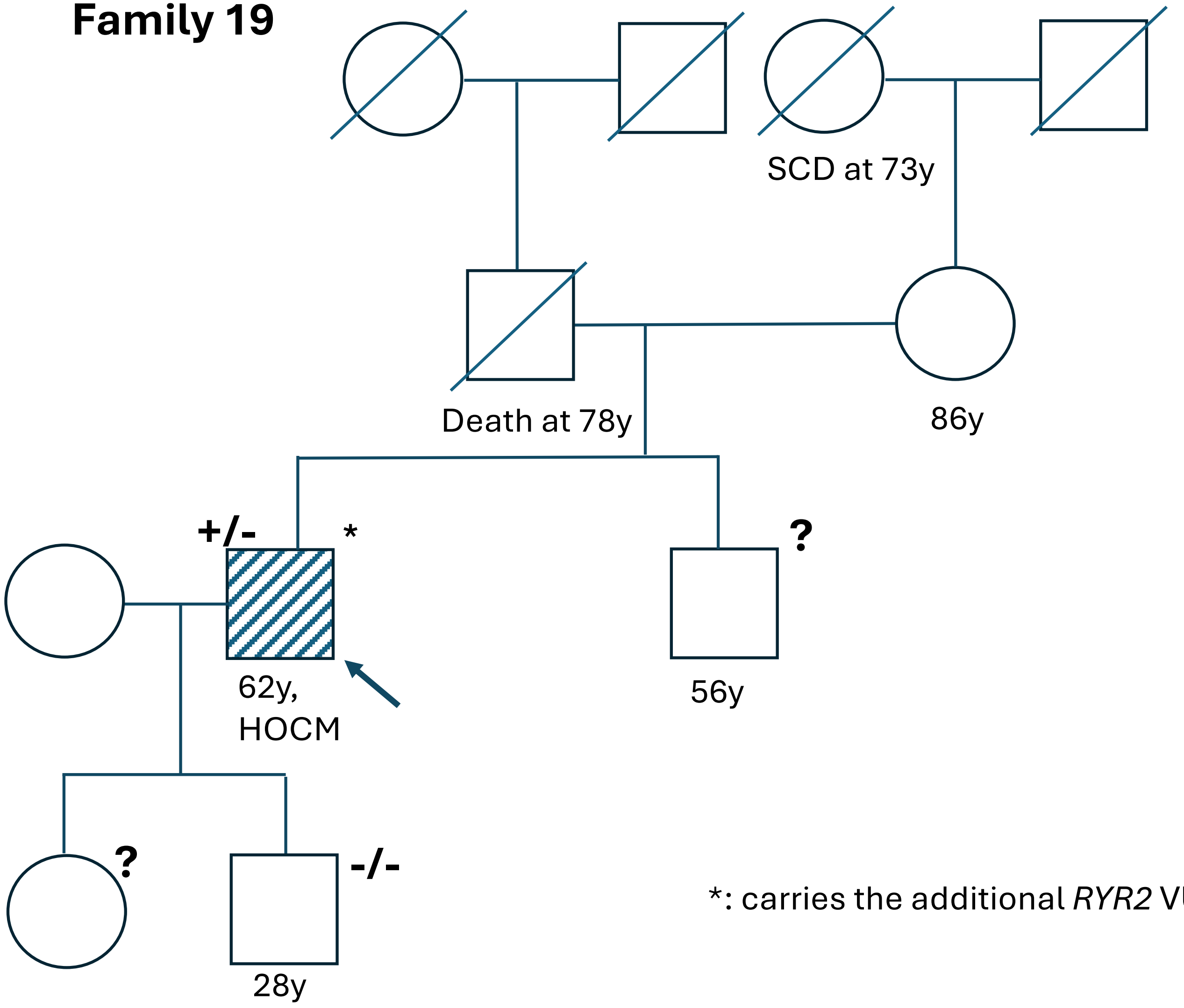


*: carries the additional *RYR2* VUS

## Family 20

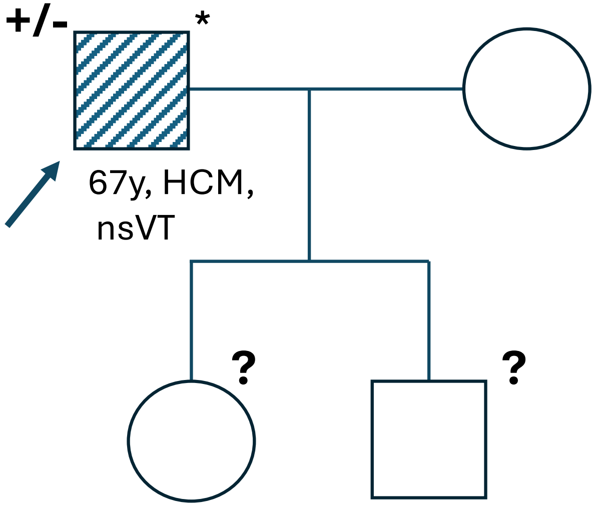


*: carries the additional *DSP* VUS

## Family 21

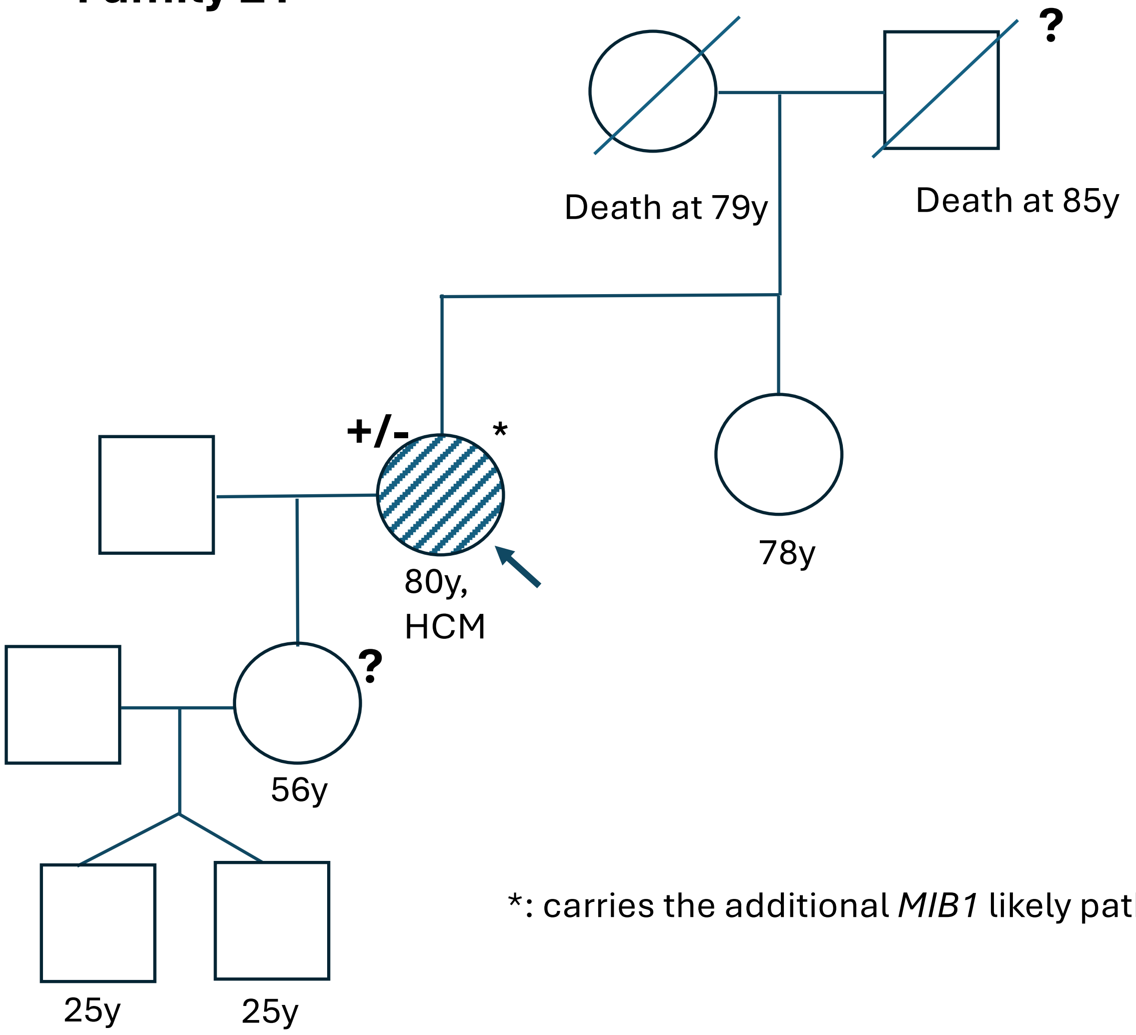


*: carries the additional *MIB1* likely pathogenic variant

## Family 22

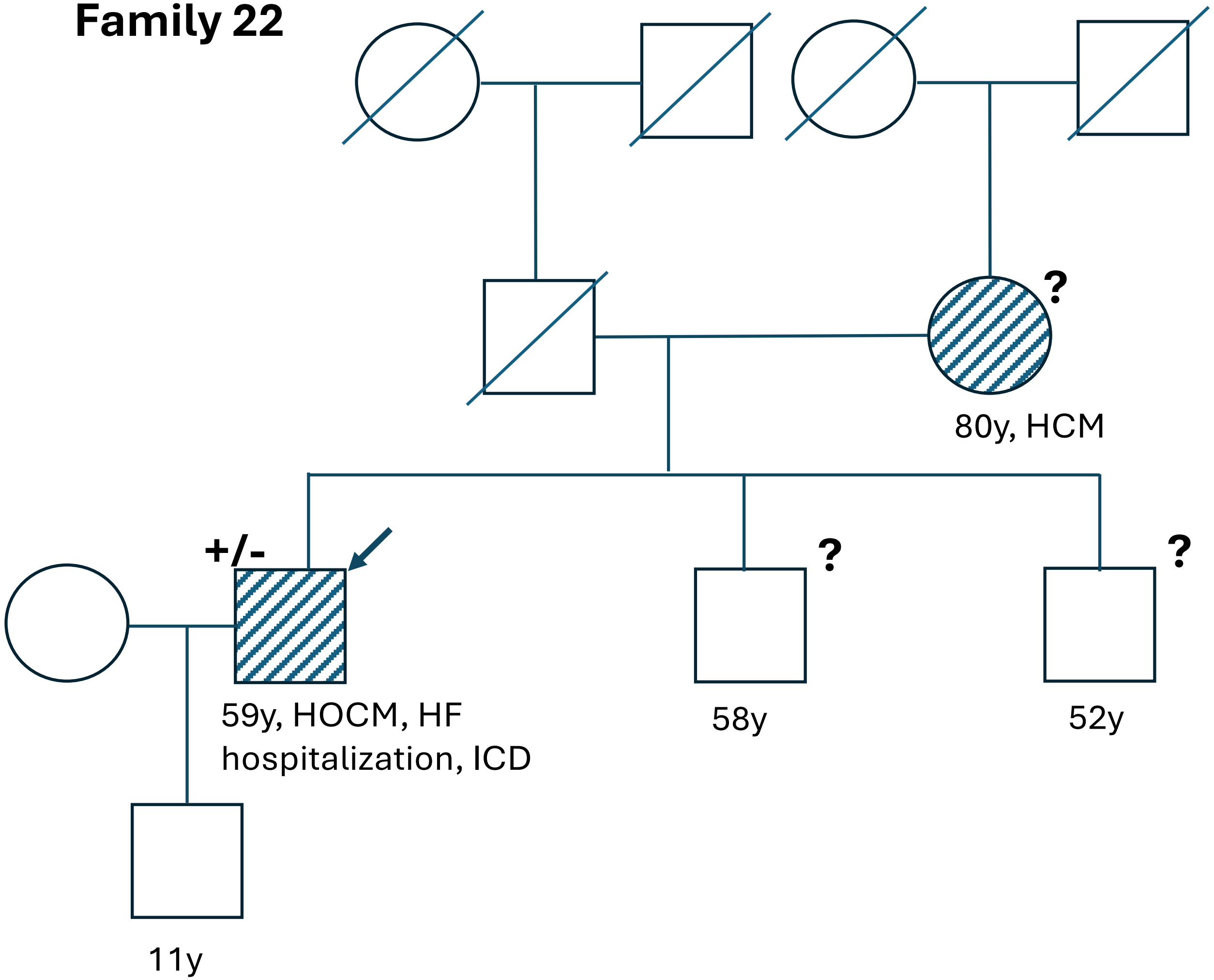

## Family 23

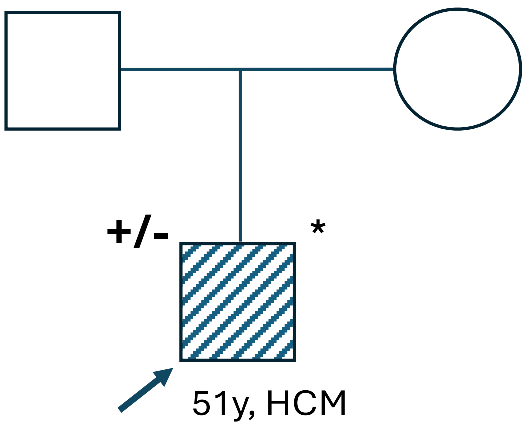


*: carries the additional *MYH7* VUS

## Family 24

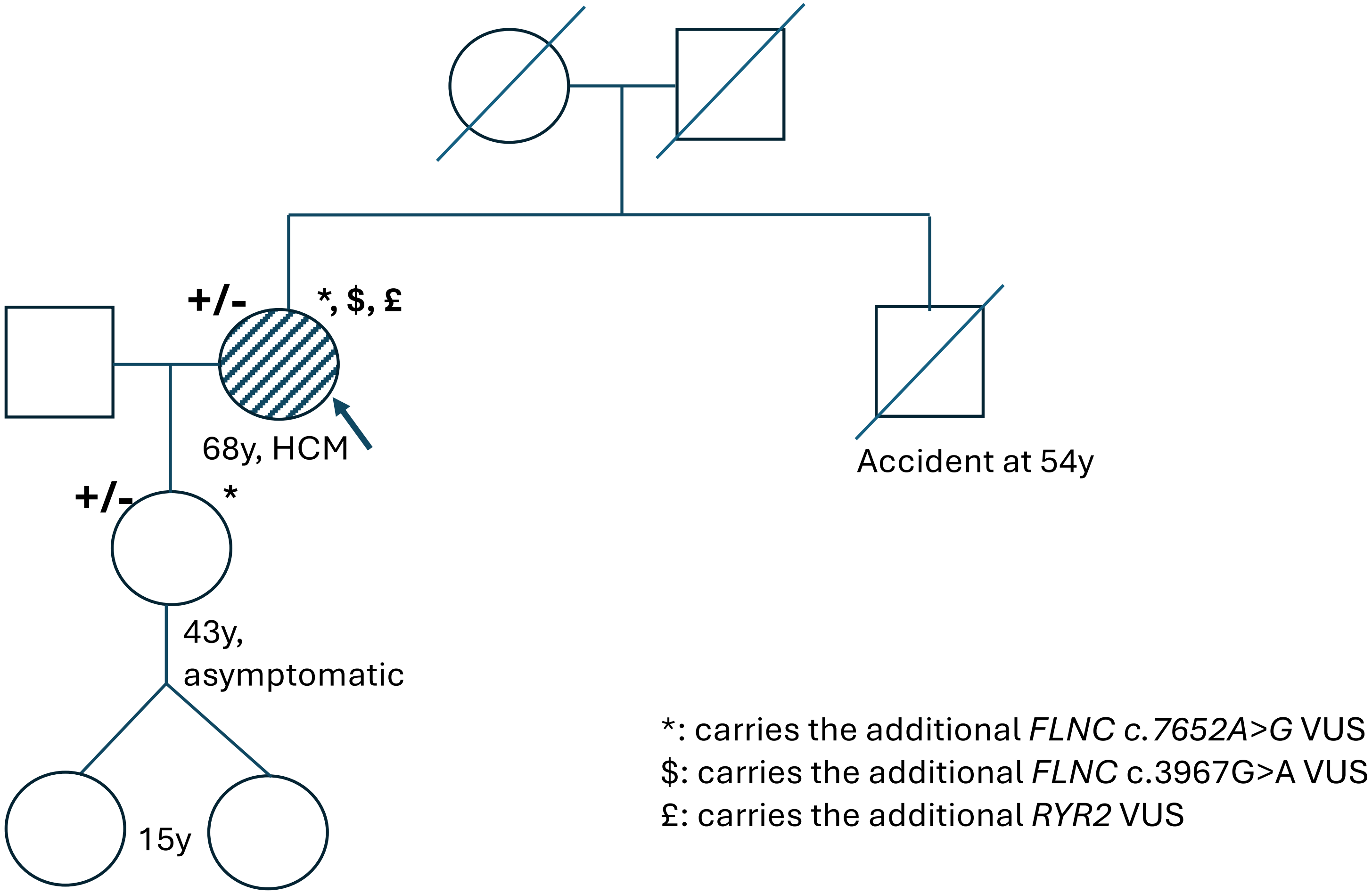
+/-
*, $, £
68y, HCM
Accident at 54y
+/-
*
43y,
asymptomatic
15y
*: carries the additional *FLNC c.7652A>G* VUS
$: carries the additional *FLNC* c.3967G>A VUS
£: carries the additional *RYR2* VUS
